# Calcium/calmodulin kinase1 and its relation to thermotolerance and HSP90 in *Sporothrix schenckii*: an RNAi and yeast two-hybrid study

**DOI:** 10.1186/1471-2180-11-162

**Published:** 2011-07-11

**Authors:** Jorge Rodriguez-Caban, Waleska Gonzalez-Velazquez, Lizaida Perez-Sanchez, Ricardo Gonzalez-Mendez, Nuri Rodriguez-del Valle

**Affiliations:** 1 Department of Microbiology and Medical Zoology, Medical Sciences Campus, University of Puerto Rico, PO Box 365067, San Juan, PR 00936-5067, USA; 2 Department of Radiological Sciences, Medical Sciences Campus, University of Puerto Rico, PO Box 365067, San Juan, PR 00936-5067, USA

## Abstract

**Background:**

*Sporothrix schenckii *is a pathogenic dimorphic fungus of worldwide distribution. It grows in the saprophytic form with hyaline, regularly septated hyphae and pyriform conidia at 25°C and as the yeast or parasitic form at 35°C. Previously, we characterized a calcium/calmodulin kinase in this fungus. Inhibitors of this kinase were observed to inhibit the yeast cell cycle in *S. schenckii*.

**Results:**

The presence of RNA interference (RNAi) mechanism in this fungus was confirmed by the identification of a Dicer-1 homologue in *S. schenckii *DNA. RNAi technology was used to corroborate the role of calcium/calmodulin kinase I in *S. schenckii *dimorphism. Yeast cells were transformed with the pSilent-Dual2G (pSD2G) plasmid w/wo inserts of the coding region of the calcium/calmodulin kinase I (*sscmk1*) gene. Transformants were selected at 35°C using resistance to geneticin. Following transfer to liquid medium at 35°C, RNAi transformants developed as abnormal mycelium clumps and not as yeast cells as would be expected. The level of *sscmk1 *gene expression in RNAi transformants at 35°C was less than that of cells transformed with the empty pSD2G at this same temperature. Yeast two-hybrid analysis of proteins that interact with SSCMK1 identified a homologue of heat shock protein 90 (HSP90) as interacting with this kinase. Growth of the fungus similar to that of the RNAi transformants was observed in medium with geldanamycin (GdA, 10 μM), an inhibitor of HSP90.

**Conclusions:**

Using the RNAi technology we silenced the expression of *sscmk1 *gene in this fungus. RNAi transformants were unable to grow as yeast cells at 35°C showing decreased tolerance to this temperature. The interaction of SSCMK1 with HSP90, observed using the yeast two-hybrid assay suggests that this kinase is involved in thermotolerance through its interaction with HSP90. SSCMK1 interacted with the C terminal domain of HSP90 where effector proteins and co-chaperones interact. These results confirmed SSCMK1 as an important enzyme involved in the dimorphism of *S. schenckii*, necessary for the development of the yeast phase of this fungus. Also this study constitutes the first report of the transformation of *S. schenckii *and the use of RNAi to study gene function in this fungus.

## Background

Pathogenic fungi use signal transduction pathways to sense the environment and to adapt quickly to changing conditions. Identification of the components that comprise signalling cascades controlling dimorphism in *Sporothrix schenckii *has been of particular interest in our laboratory for years. Studying the mechanisms controlling dimorphism in *S. schenckii *is important for understanding its pathogenicity and the response to the hostile environment encountered in the host [1,2]. Dimorphism in *S. schenckii *as in other pathogenic fungi has been associated with virulence [3,4]. This fungus exhibits mycelium morphology in its saprophytic phase at 25°C and yeast morphology in host tissues at 35-37°C. Studies on the role of calcium in *S. schenckii *dimorphism showed that calcium stimulates the yeast to mycelium transition and that calcium uptake accompanies this transition [5].

Calcium is one of the most important intracellular second messengers and is involved in a wide range of cellular events in many eukaryotic cells [6,7]. Calcium can affect cellular processes by binding to calmodulin (CaM) that in turn activates Ca**^2+^**/calmodulin-dependent protein kinases (CaMKs) [8-10]. These serine/threonine protein kinases have two major domains: a highly conserved amino-terminal catalytic domain and a carboxy-terminal regulatory domain. The regulatory domain consists of the autoinhibitory and Ca^2+^/CaM binding domains. The autoinhibitory domain acts as a pseudosubstrate, blocking access to the catalytic site [11]. Ca^2+^/calmodulin binding to the regulatory domain causes a conformational change in Ca^2+^/CaM kinases exposing the catalytic domain by removing the autoinhibitory domain. This enables the binding of the substrate and its subsequent phosphorylation [9,11].

The Ca^2+^/calmodulin kinases constitute a family of related kinases that includes CaMKK, myosin light chain kinase and CaMKI to CaMKIV. The role of CaMKs in mammalian systems, particularly in neurons is well established [12], while their presence and role in fungi is not fully documented. CaMKs have been described for *Saccharomyces cerevisiae *[13], *Aspergillus nidulans *[14-17], *Schizosaccharomyces pombe *[18] and *Neurospora crassa *[19], among others. Whole genome sequencing projects also show the presence of hypothetical proteins homologous to CaMK in many other fungi. In *S. cerevisiae*, the CaMKs function in the survival of pheromone-induced growth arrest, salt tolerance and thermotolerance [20]. In the filamentous fungus *A. nidulans*, the disruption of the CaMK encoding genes, CMKA and CMKB was reported to be lethal [14,15]. In this fungus, CaMK is required for progression through the nuclear division cycle [16].

In *S. schenckii*, we described a CaMK encoded by the *sscmk1 *gene (GenBank accession no. AY823266) [21]. The SSCMK1 cDNA encoded a protein of 407 amino acids with a calculated molecular weight of 45.6 kDa. The analysis of the derived amino acid sequence revealed a calcium/calmodulin kinase containing the 12 conserved sub-domains necessary for a functional serine/threonine protein kinase [22] and a serine/threonine protein kinase catalytic domain. Experiments using three different inhibitors of the CaMK pathway, W-7, KN-62 and lavendustin C [23-27], showed that they inhibited the re-entry of yeast cells into the budding cycle [21]. This observation was the first evidence of the involvement of a calcium/calmodulin pathway in the regulation of dimorphism in *S. schenckii *[21].

Traditionally, gene function analysis have been performed by examining the phenotypic or biochemical changes observed in organisms harbouring a mutation in the gene of interest or by gene knockout studies [28]. In this respect *S. schenckii *has been considered a genetically intractable organism. In the case of *S. schenckii *no successful transformation protocol has been implemented. In many other fungi, the transformation process has proven laborious, time-consuming and has potential disadvantages such as non-homologous recombination. Alternatively, RNA-mediated gene silencing has been used to manipulate gene expression in eukaryotic organisms and fungi [29-32]. In fungi, RNA-mediated gene silencing has been demonstrated in many species [31]. To date, there are no reports of the use of RNAi for the study of gene function in *S. schenckii*.

In this work we provide evidence of the presence of the RNAi mechanism in *S. schenckii *by identifying a key enzyme of the RNAi system, a DCL-1 homologue. We show that *S. schenckii *can be successfully transformed. We also knocked down the expression of the *sscmk1 *gene in *S. schenckii *using RNAi. Transformed cells exhibited an inhibition in the development of the yeast phase, which coincides with our previous report that SSCMK1 is needed for the expression of the yeast morphology. Yeast two-hybrid analysis of proteins interacting with SSCMK1 showed the interaction of this enzyme with a HSP90 homologue, a very important player in fungal thermotolerance. Inhibiting SSHSP90 with geldanamycin (GdA) also inhibited the development of the yeast form of the fungus and the growth observed was similar to that obtained with the SSCMK1 RNAi transformants.

## Results

### Presence of a Dicer-1 homologue in *S. schenckii *DNA

**Figure 1:**
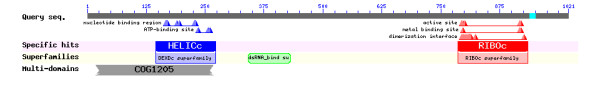
**Protein domains analysis of *S. schenckii *DCL-1 homologue**. This figure shows 3 of the 4 domains that characterize the Dicer-1 proteins that were present in the *S. schenckii *DCL-1 homologue fragment. The domains were identified using the NCBI Conserved Domain Database. The domains in the 1021 amino acid fragment were: HELIC_c (helicase domain), dsRNA binding and the RIBOc domains.

A PCR homology approach was used to identify a Dicer-1 homologue in *S. schenckii *DNA. Figure 1 shows the conserved domains detected in this protein fragment using the NCBI Conserved Domain Database. Sequence analysis shows 3 characteristic domains of the DCL proteins: a helicase C domain, a dsRNA binding domain and an RNAse 3 domain. This PCR product (GenBank accession numbers: GQ414744.1 and ACU45742.1 for the genomic and amino acid sequence, respectively) shows a 3140 bp fragment, encoding 1021 amino acids, corresponding to a central, inner fragment of a dicer-1 protein homologue (Additional File 1). This sequence includes a putative intron from nucleotide 2163 to nucleotide 2237 because genomic DNA was used as template for PCR. An intron is also present in the *N. crassa *gene in this position. The Panther Classification System identified this protein as a member of a yet to be named family of proteins comprised of the *N. crassa *and the *Schizosaccharomyces pombe *ATP dependent helicase DCL-1 with an E value of 5.5 e^-208^.

Additional File 2 shows the amino acid sequence alignment of the SSDCL-1 fragment to other fungal DCL-1 homologues. This alignment shows that these proteins are highly conserved among fungi, specifically in the regions of the above mentioned domains.

### Transformation of *S. schenckii*

A method for the transformation of *S. schenckii *was successfully implemented based on a modification of the method of Royer *et al*. [33], for other *Ophiostomaceae*. This method was chosen after testing various transformation methods with *S. schenckii *yeast cells. Two transformations were done, one using pSD2G and pSD2G-RNAi1 and the other using pSD2G and pSD2G-RNAi2 (Additional File 3A and 3B). For the first transformation, yeast cells were grown from conidia to a concentration of 10^9 ^cells/ml as described previously, in a modification of medium M. These logarithmically growing cells were converted to protoplasts as described in Methods. The number of cells converted to protoplasts in the first transformation was 76%. The protoplasts were not separated from the undigested cells in order to avoid further damage to these cells. The cells were divided into 3 groups, each containing 200 μl of the suspension. The cells in the first group were treated with non-transforming DNA. In the second group, cells were transformed with pSD2G (Additional File 3A) and in the last group; the cells were transformed with pSD2G-RNAi1 (Additional File 3A). Two hundred and twelve colonies were obtained from the cells transformed with pSD2G and 242 colonies were obtained from cells transformed with pSD2G-RNAi1. Transformants were transferred to fresh geneticin-containing medium and grown for 5-10 days in medium M plates at 35°C. Ninety five percent of the colonies transformed with pSD2G and 97% of those transformed with pSD2G-RNAi1 survived transfer under these same conditions.

For the second transformation the same protocol was used. Seventy nine percent of the cells transformed with pSD2G-RNAi2 (Additional File 3B) survived transfer to fresh geneticin-containing medium. Conidia from transformants surviving this passage were used to inoculate 50 ml of medium M with geneticin (500 μg/ml) at 35°C with aeration.

Further passages decreased the number of the RNAi transformants capable of growing at 35°C. These cultures, where no growth was detected at 35°C, were transferred to 25°C and all of them thrived, showing mycelium morphology in spite of their inability to grow at 35°C.

Additional File 3C also shows the results of colony PCR used to detect the presence of the transforming DNA in *S. schenckii *yeast cells transformed with pSD2G-RNAi1. Cell suspensions of *S. schenckii *transformants were used as templates for PCR using the G418 (fwd) and G418 (rev) primer pair. Lane 4 shows the 123 bp DNA ladder. Lanes 1-5 and 6 shows the bands obtained when the cells transformed with pSD2G-RNAi1 from colonies 14, 15, 18, 19 and 21 were used as template, respectively. In lanes 7 and 8, suspensions of non-transformed cells were used as templates for PCR. A band of the expected size, 622 bp, detecting the presence of the geneticin resistance cassette was observed in transformed yeast cells.

### Morphology of transformed cells

**Figure 2:**
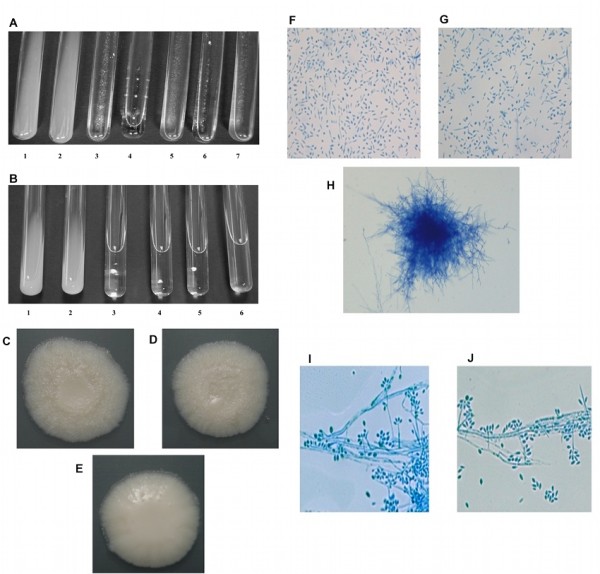
**Macroscopic and microscopic appearance of *S. schenckii *transformants and controls incubated at 35°C and 25°C**. Figures 2A and 2B show the appearance of *S. schenckii *transformed with pSD2G, pSD2G-RNAi1 or pSD2G-RNAi2 grown in liquid medium w/wo geneticin (500 μg/ml) and incubated at 35°C. In Figure 2A, tube 1 shows the growth of the wild type cells (no geneticin added to the medium), tube 2 shows the growth of cells transformed with the empty plasmid (pSD2G). Tubes 3 to 7 show the growth obtained from colonies 19, 21, 29, 33 and 47, respectively that were transformed with pSD2G-RNAi1. In Figure 2B, tubes 1 and 2 show the growth observed with the wild type cells and cells transformed with the pSD2G, respectively. Tubes 3 to 6 show the growth obtained from colonies 1, 2, 7 and 16, transformed with pSD2G-RNAi2. Figure 2C, 2D and 2E show the appearance of *S. schenckii *transformed with pSD2G or pSD2G-RNAi1 grown in solid medium w/wo geneticin (500 μg/ml) and incubated at 25°C. Figure 2C shows the growth of cells transformed with pSD2G. Figure 2D and 2E show the growth obtained from colonies 19 and 21 transformed with pSD2G-RNAi1, respectively. Figure 2F, 2G and 2H show the microscopic morphology of wild type and transformed cells of *S. schenckii *grown from conidia as described in Methods for 5 days at 35°C in liquid medium w/wo geneticin (500 μg/ml) and mounted on lactophenol cotton blue. Samples F and G correspond to the wild type and cells transformed with pSD2G respectively, at 40× magnification. Sample H shows the appearance of cells transformed with the *sscmk1 *pSD2G-RNAi1 at 20× magnification. Figure 2I and 2J show the microscopic morphology on slide cultures of *S. schenckii *grown from conidia as described in Methods at 25°C in solid medium w/wo geneticin (500 μg/ml) and mounted on lactophenol cotton blue of cells transformed with pSD2G and cells transformed with pSD2G-RNAi1, respectively.

Conidia from cells transformed with pSD2G or pSD2G-RNAi1 were inoculated in liquid medium with geneticin (500 μg/ml) and incubated at 35°C, distinct differences were observed between the growth of cells transformed with pSD2G and those transformed with pSD2G-RNAi1. The cells transformed with pSD2G grew as abundantly as the wild type cells with the appearance of yeast cell growth, while the cells transformed with pSD2G-RNAi1 showed little growth, resembling mycelia, a morphology not observed at 35°C (Figure 2A). Tube 1 shows the growth observed in wild type cells, tube 2 shows the growth observed in cells transformed with the empty plasmid pSD2G and tubes 3 to 7 show the growth obtained from colonies 19, 21, 29, 33 and 47, respectively, transformed with pSD2G-RNAi1.

A second transformation using pSD2G-RNAi2 corroborated the phenotypic changes observed with the 3' fragment insert (pSD2G-RNAi1) and served as evidence that the observed morphological changes when using pSD2G-RNAi1 for transformation were not due to off-target effects. The same morphology was obtained when the fragment cloned into pSD2G was from the 5' end of the *sscmk1 *gene (pSD2G-RNAi2) as shown in Figure 2B. Tubes 1 and 2 show the growth observed with the wild type cells and cells transformed with the empty plasmid, respectively. Tubes 3 to 6 show the growth obtained from colonies 1, 2, 7 and 16, respectively, transformed with pSD2G-RNAi2.

Transformants, even those that could not grow at 35°C, developed into mycelia and grew almost as abundantly as the wild type at 25°C. Figure 2 shows samples of the mycelial growth obtained in agar plates of a modification of medium M with geneticin at 25°C. Figure 2C corresponds to the growth observed in cells transformed with pSD2G and Figure 2D and 2E correspond to the growth observed from colonies 19 and 21 transformed with pSD2G-RNAi1, respectively.

### Microscopic morphology of transformed cells

The microscopic observation of the cultures mentioned above in Figure 2A revealed that wild type cells and cells transformed with pSD2G grew as yeasts at 35°C as shown in Figure 2F and 2G, respectively. The cells transformed with pSD2G-RNAi1 showed clumps of mycelia and very few yeast cells when compared to the controls (Figure 2H) at this same temperature.

Figure 2 also shows the morphology on slide culture of mycelia that developed from conidia produced by pSD2G (Figure 2I) and pSD2G-RNAi1 transformants (Figure 2J) in a modification of medium M with agar and geneticin at 25°C. No differences were observed in the appearance of the mycelia or in conidiation between cells transformed with pSD2G and those transformed with pSD2G-RNAi1 at 25°C.

### Quantitative Real-Time RT-PCR

**Figure 3:**
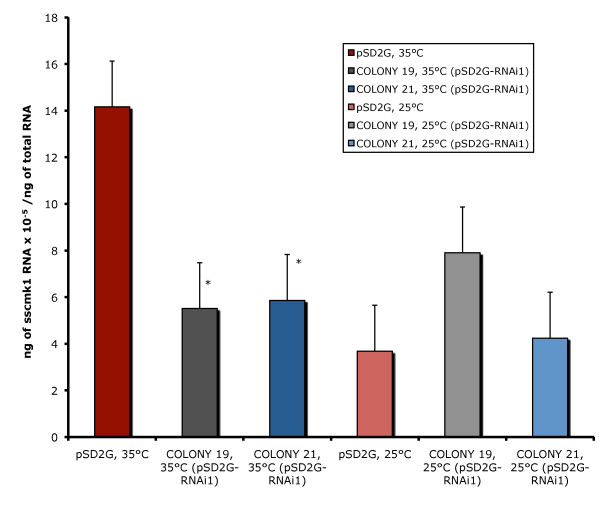
**Analysis of the expression of *sscmk1 *RNA in *S. schenckii *cells transformed with pSD2G or pSD2G-RNAi1 grown at 35°C and 25°C**. The expression of *sscmk1 *gene RNA was determined in cells transformed with plasmid pSD2G and plasmid pSD2G-RNAi1. RNA was extracted as described in Methods from cells growing in a modification of medium M with geneticin (500 μg/ml) at 35°C or cells growing in a modification of medium M with geneticin (500 μg/ml) at 25°C. A minimum of 3 independent experiments were performed for each transformant. The average ± the standard deviation of the ng of *sscmk1 *RNA/ng of total RNA was calculated using the standard curve. The Student's T test was used to determine the significance of the data (p < 0.05). Results significantly different from the control values are marked with an asterisk.

Figure 3 shows the results obtained using quantitative real time RT-PCR (qRT-PCR) of cells transformed with pSD2G and pSD2G-RNAi1. This figure shows that the cells transformed with pSD2G-RNAi1 and incubated at 35°C had approximately 60% less *sscmk1 *RNA than those transformed with pSD2G and that these differences were significant (p < 0.05). These results suggest that the levels of *sscmk1 *transcript must increase for yeast cells to develop at 35°C. The cells transformed with pSD2G-RNAi1 cannot attain this level of *sscmk1 *RNA and they grow poorly as mycelia at 35°C. The *sscmk1 *RNA of these same cells grown as mycelia at 25°C is lower and no significant differences were observed in cells transformed with the empty plasmid (pSD2G) and those transformed with pSD2G-RNAi1.

### Yeast two-hybrid assay

More than 25 inserts from colonies growing in quadruple dropout medium (QDO) (SD/-Ade/-His/-Leu/-Trp) from two different *S. schenckii *yeast cDNA libraries were analyzed for the presence of SSCMK1 interacting proteins. Only inserts from colonies that grew in QDO were cloned and sequenced. Two different inserts were identified as belonging to a homologue of HSP90. The sequence obtained by PCR from one of these inserts showed a 778 bp product and a derived amino acid sequence of 164 amino acids of the C-terminal domain of this protein. The other insert contained 477 bp and encoded the last 64 amino acids of the protein.

**Figure 4:**
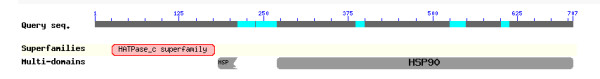
**Protein domains analysis of *S. schenckii *HSP90 homologue**. This figure shows the domains that characterize the HSP90 homologue of *S*. *schenckii*. The domains were identified using the NCBI Conserved Domain Database. The domains in the 707 amino acid protein were: HATPase_c (histidine kinase ATPase domain) and the HSP90 domains.

Figure 4 shows the conserved domains detected in this protein using the NCBI Conserved Domain Database. Sequence analysis identified a HATPase_c and the HSP90 domains. Using the RACE technique, we obtained an open reading frame of 2121 nucleotides encoding a HSP90 homologue of 707 amino acids with an estimated molecular weight of 80.17 kDa. Pfam identified this sequence as belonging to heat shock protein 90 with an E value of 5.8 e^-255^. The GenBank accession numbers are JF412349.3 and AEA51002.2 for the cDNA and amino acid sequence, respectively.

The complete coding cDNA sequence of SSHSP90 is shown in Additional File 4. In this figure, amino acid residues involved in the interaction with tetratricopeptide repeat proteins are shown in red letters and the HATPase domain is shaded in yellow.

Additional file 5 shows the multiple sequence alignment of various fungal HSP90 and the human HSP90 isoform 2. This figure shows the high degree of conservation of HSP90 fungal homologues, including SSHSP90. The HATPase or N terminal domain region is boxed in blue while the HSP90 domain region is boxed in red. A blue line marks the C terminal domain.

**Figure 5:**
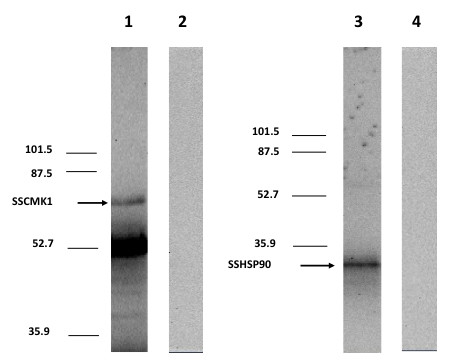
**Co-Immunoprecipitation and Western Blot of SSCMK1 and HSP90**. This figure shows the results obtained with co-immunoprecipitation and Western Blot analysis of SSCMK1 interacting with SSHSP90.Whole cell free extracts of *S. cerevisiae *cells expressing the complete c-myc tagged SSCMK1coding sequence fused to the GAL4 activation domain (bait protein) and the HA tagged protein fragment fused to the GAL4 DNA binding domain (prey protein) were co-immunoprecipitated as described in Methods. The co-immunoprecipitated proteins were separated using 10% SDS polyacrylamide electrophoresis and transferred to nitrocellulose. The nitrocellulose strips were probed with anti-cMyc antibodies (Lane 1) and anti HA antibodies (Lane 3). Pre-stained molecular weight markers were included in outside lanes of the gel. The position of the molecular weight markers is indicated in the figure. Lanes 2 and 4 are negative controls where no primary antibody was added.

Figure 5 shows the confirmation of the interaction of SSCMK1 with the HSP90 homologue using co-immunoprecipitation (Co-IP) and Western blot. The Co-IP's result for SSCMK1 shows a band of 71 kDa. The calculated theoretical value, considering that SSCMK1 was expressed fused to the GAL-4 binding domain is 68 kDa. The lower band observed in Lane 1 corresponds to the heavy chain of the antibody used for Co-IP. Lane 2 shows the results obtained in the Western blot when the primary anti-cMyc antibody was not added (negative control). Lane 3 shows the band obtained using anti-HA antibody that recognizes the SSHSP90 fragment. The observed molecular weight of this band is 33.0 kDa. This molecular weight is within the expected value considering that this fragment is fused to the GAL-4 activation domain (the theoretical value is 36 kDa). Lane 4 shows the results obtained in the Western blot when the primary anti-HA antibody was not added (negative control). The differences between the observed and the theoretical molecular weight could be due to sodium dodecyl sulfate (SDS) binding and could also be the effect of post-translational modifications of the peptides including phosphorylation.

**Figure 6:**
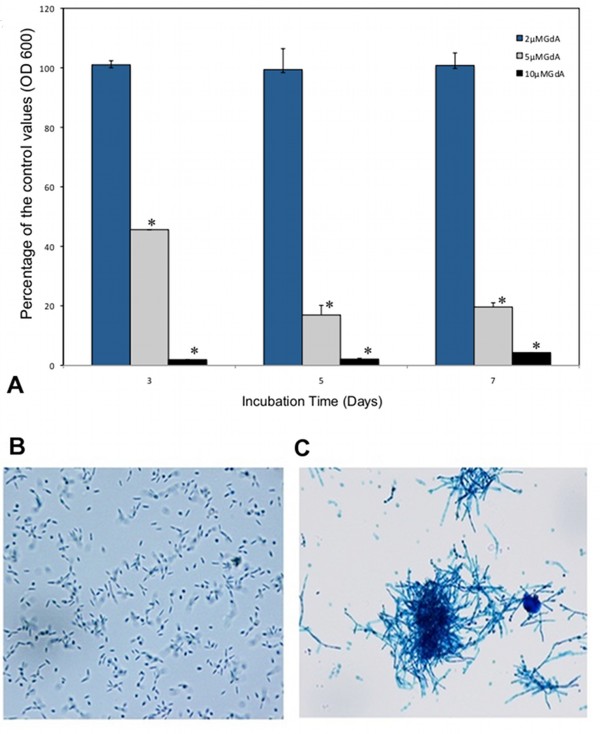
**Effects of geldanamycin on growth and morphology**. *S. schenckii *conidia (10^9^) were inoculated in a modification of medium M containing 2, 5 and 10 μM concentrations of geldanamycin. The growth was recorded as OD at 600 nm at 3, 5 and 7 days of incubation as described in Methods. The percentage of growth of the *S. schenckii *in the presence of geldanamycin when compared to that of the controls of 3 independent experiments is given ± a standard deviation. Values significantly different from the controls are marked with an asterisk. Samples of the growth obtained after 7 days at 35°C in liquid medium w/wo geldanamycin (10 μM) were drawn and mounted on lactophenol cotton blue. Figure 6A corresponds to the controls cells at 40× magnification. Figure 6B shows the appearance of cells grown in the presence of geldanamycin at 20× magnification. Microscopic observations of the fungus were done using a Nikon Eclipse E600, equipped with a Nikon Digital Sight DS-2Mv and the NIS-Elements F 2.3 software.

Figure 6A shows the effects of different concentrations of geldanamycin (GdA), an inhibitor of HSP90 on the development of conidia into yeast cells at 35°C. This figure shows a significant inhibition of growth at concentrations of 5 and 10 μM GdA using multiple comparison Student's T test (p < 0.05). This suggests that HSP90 is needed for yeast cells growth at 35°C. Figure 6B shows the microscopic morphology of cells grown in the presence of GdA (10 μM) and that of the controls after 7 days of incubation. The control cells (Figure 6B) show normal yeast morphology while the cells growing with 10 μM GdA (Figure 6C) added to the medium showed a morphology similar to that of the cells transformed with pSD2G-RNAi1 shown in Figure 2H.

## Discussion

Implementing a suitable transformation system that would be effective for *S. schenckii *was one of our main goals. Gene knockout studies in *S. schenckii *have been hindered by two main reasons: first, the fungus is possibly diploid and second, no suitable transformation system has proven useful for this fungus. The information suggesting that *S. schenckii *is diploid comes from early studies done by us comparing the DNA content of our strain (μg of DNA/cell) with that of a diploid *Candida albicans *and haploid *S. cerevisiae*. In these experiments the DNA content of our strain was similar to that of the diploid *C. albicans *and to twice that of the haploid *S. cerevisiae *(unpublished results). If our S*. schenckii *strain is diploid, one would have to effectively knockout both copies of a given gene using 2 markers to select the transformants.

A variety of transformation systems have been developed for many fungi, being the most popular that of Ito and collaborators for *S. cerevisiae *[34]. Preliminary work done by us using this method showed that this transformation protocol was not useful for *S*. *schenckii *yeast cells (unpublished results). In this paper we describe the adaptation of a method originally designed for the transformation of *Ophiostoma ulmi *by Royer *et al*., for the transformation of *S. schenckii *[33]. This method uses permeabilized cells and treatment with β-mercaptoethanol, both of these conditions have been observed by us to increase the success of transformation of *S. schenckii*, as is the case of *Ophiostoma ulmi *[33].

The frequency of transformation for all fungi is dependent on a variety of different parameters such as the nature of the transforming DNA, the concentration of the transforming DNA and the selection agent, among others [34-36]. Our primary goal in this work was to obtain the greatest number of transformants; therefore a concentration of transforming DNA of the order of 10 μg per 10^8 ^cells was used. Having used this amount of DNA, a frequency of transformation of approximately 24 transformants/μg of DNA was obtained. This number of transformants is within the range reported with other fungi specifically when unlinearized DNA is used [34].

After having a reliable transformation system for *S. schenckii*, the next goal was to inquire if RNAi was an option to study gene function in this fungus. Due to the uncertainty as to the presence of the gene silencing mechanism in some fungi such as *S. cerevisiae *and *Ustilago maydis *[37], we identified the presence of one of the enzymes involved in processing RNAi in *S. schenckii *DNA, a Dicer-1 homologue. As stated previously, the Dicer enzymes are important components of the mechanism that processes double stranded RNA precursors into small RNAs [38]. In the filamentous fungi, one or two Dicer-like homologues have been described [39-41]. *N. crassa *is the fungus where quelling was first described and has been more thoroughly studied [42]. In this fungus two Dicer-like homologues, *dcl-1 *and *dcl-2 *genes have been described [39]. The double mutant *dcl-1 *and *dcl-2 *showed the suppression of the processing of dsRNA into siRNA in *N. crassa*.

Having validated the presence of the RNAi processing mechanism and having a suitable transformation system for *S. schenckii*, the *sscmk1 *gene was targeted using RNAi directed to knockdown the expression of this gene. *S. schenckii *yeast cells were first transformed with pSD2G-RNAi1 containing a segment of the 3' end of the *sscmk1 *gene. The size of the *sscmk1 *insert used for transformation was in the range used for other fungal RNAi transformations [43,44]. Real-time PCR (qRT-PCR) confirmed that the levels of *sscmk1 *transcript were lower for the cells transformed with the pSD2G-RNAi1 than for the cells transformed with the empty plasmid at 35°C.

The pSD2G-RNAi1 transformants grew from the beginning as mycelium type colonies in the selection plates at 35°C. Later when cultivated in liquid medium with aeration at 35°C, the growth observed, if any, was scarce and had the appearance of mycelium clumps with very few yeast cells. Upon further transfers to fresh medium, some of the conidia lost the capacity to grow at 35°C but could grow as mycelia when these same cultures were transferred to 25°C, as stated previously. The inability to grow at 35°C could be due to a gradual lowering of the intracellular SSCMK1 levels and the resulting impairment of thermotolerance in these cells, not viability. The fact that the conidia from some pSD2G-RNAi1 transformants could not grow at 35°C but if transferred to 25°C developed into mycelia and grew almost as abundantly as the wild type reinforces our previous results that suggest that SSCMK1 is necessary for the development of the yeast form of the fungus.

In order to dismiss the possibility that the morphological effects could be due to an off-target effect, a second transformation was done using a different insert, this time from the 5' end of the *sscmk1 *gene. The same abnormal morphology and growth at 35°C was observed when pSD2G-RNAi2 was used for transformation.

The growth phase affected by silencing the *sscmk1 *gene was that of the yeast form of the fungus. In *S. schenckii*, the development of the yeast form of this fungus is favoured by increasing the temperature to 35°C. The capacity to tolerate temperatures between 35-37°C is essential for *S. schenckii *to grow in the human host. Some other species of the *Ophiostomaceae *that are plant pathogens, can produce yeast cells but most lack the ability to grow at 35-37°C and are non-pathogenic to humans [1].

Previous results using CaMK inhibitors pointed to the role of SSCMK1 for the proliferation of the yeast cells induced to re-enter the cell cycle and for the maintenance of the yeast morphology in *S. schenckii*. In this work, we observed these same results but we also observed that the actual effect could lie in the loss of thermotolerance by the fungus when *sscmk1 *was silenced.

CaM kinases in many systems, including fungi, have been reported to have an effect in the control of the cell cycle, differentiation and/or gene expression, specifically through the activation of transcription factors [45-47]. At the time of our first report, we hypothesized that SSCMKI was needed for the phosphorylation of proteins involved in the regulation of the cell cycle and/or for the phosphorylation and activation of transcription factors needed for the dimorphic transitions of the fungus. However, we mentioned that the final interpretation of our results awaited the identification of the interacting partners of SSCMKI that was also accomplished in this work.

Important information related to the role of SSCMK1 in *S. schenckii*, was obtained with the yeast two-hybrid assay. Among the many proteins identified as interacting with SSCMK1 we identified a *S. schenckii *homologue of HSP90. This interaction was corroborated with Co-IP. It is a well-known fact that all organisms from bacteria to higher eukaryotes respond to elevated temperatures by producing heat shock proteins. Two important observations regarding a connection between the heat shock response and CaMKs have been reported. In *C. albicans*, this kinase was shown to have a role in the capacity of fungal cells to grow at elevated temperature [48] and in *Arabidopsis thaliana*, CaMK-3 has been observed to be part of the heat shock response, possibly by the phosphorylation of the heat shock response factor and the induction of the transcription of the heat shock proteins [49]. In tomato (*Solanum lycopersicum*), LeCPK2, a CaMK, is up regulated in response to heat stress [50].

Heat shock proteins are a widespread family of molecular chaperones found in bacteria and all eukaryotic organisms. These chaperones ensure both the folding of newly synthesized proteins and their refolding under denaturing stress conditions [51]. HSP90 has been reported to interact with protein kinases. Specifically during the cell cycle, HSP90 has been reported to intervene, together with cdc37, in the stabilization of the monomeric cdk4, prior to its interaction with cyclin D [16]. It has also been reported to interact with the protein phosphatase, calcineurin that dephosphorylates CaMKs [52,53].

The interaction of HSP90 with protein kinases occurs at the N terminal domain of the HSP and two hypotheses has been postulated regarding the role of this HSP in the activity of protein kinases. HSP90 could facilitate the activation of the protein kinases by the induction of a conformational change in these kinases or could maintain the phosphorylated kinases sequestered until needed [52]. Nevertheless, SSCMK1 binds to the C terminal domain of SSHSP 90 where effectors of this heat shock protein interact. This domain starts with amino acid D621 in the human homologue of HSP90. This suggests that instead of HSP90 regulating SSCMK1, the kinase could in some form or another be regulating HSP90. If this were correct, lowering the levels of SSCMK1 would affect the function of HSP90 and in turn render the cells intolerant to high temperatures as was observed by us.

**Figure 7:**
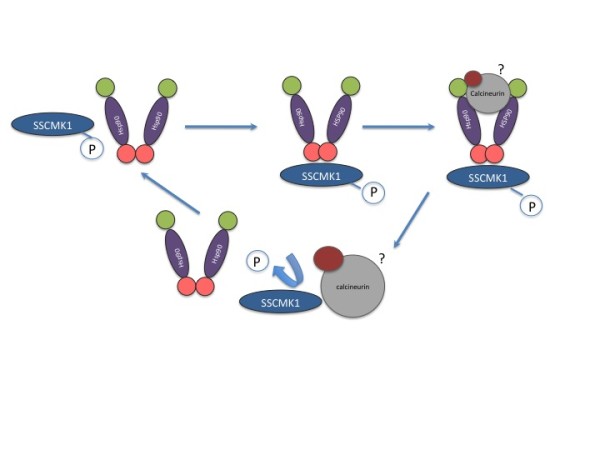
**Possible interaction of HSP90 and SSCMK1**. Evidence from RNAi inhibition of SSCMK1, HSP90 inhibition with GdA and yeast two-hybrid assay presented in this work suggests that SSCMK1 could affect fungal thermotolerance by its interaction with SSHSP90. SSCMK1 was found to interact with the C terminal domain of SSHSP90, where effectors of this heat shock protein interact. HSP90 has been identified as interacting with phosphatase, calcineurin and other kinases in many other fungal systems. The interaction of HSP90 with these proteins involves the N terminal domain. The interaction of HSP90 with calcineurin would in turn modulate the activity of SSCMK1. The presence and interaction of calcineurin in *S. schenckii *is at the moment expeculative because this protein has not been described in this fungus.

Based on this observation, we assumed that inhibitors of HSP90 should have similar effects on the growth of *S. schenckii *as was observed for pSD2G-RNAi1 and pSD2G-RNAi2 transformants. One of the most important inhibitor of HSP90 is geldanamycin. This compound was used to inhibit HSP90 in *C. albicans *where it induced yeast cells to undergo a switch to filamentous growth [48]. In *S. schenckii*, at a concentration of 10 μm, this compound induced the development of conidia into an abnormal mycelial morphology very similar to that observed in the pSD2G-RNAi transformants, at conditions suitable for the development of the yeast morphology. This is in accordance with the observation that SSCMK1 might be needed for the correct functioning of HSP90 and thermotolerance in the *S. schenckii*. Further testing using the yeast two-hybrid assay will help us identify if calcineurin is also interacting with HSP90 in *S. schenckii*, as has been reported in other fungi such as *C. neoformans *and *C. albicans *[53-55]. If this is so, we could postulate that CaMK1 regulates HSP90, and HSP90 in turn regulates CaMK1 by its effects on calcineurin and that these interactions are needed for thermotolerance in this fungus. A possible model for the interaction of HSP90 and SSCMK1 is included in Figure 7. In this figure we propose that SSCMK1 binds to HSP90 at its C terminal and this activates HSP90 and the release of effector proteins that bind to its N terminal domain, one of which can be calcineurin that can dephosphorylate the SSCMK1 and inhibit its activity. It can also release other kinases that are also effectors of fungal dimorphism. In this figure the interactions regarding calcineurin are speculative although the interaction has been reported in *C. neoformans*, this protein has not been identified in *S. schenckii *[53]

## Conclusions

The present study provides new evidence regarding the role of SSCMK1 in the development of the yeast form of *S. schenckii*. The knockdown of the *sscmk1 *gene expression using RNAi inhibited the growth of the yeast form of the fungus at 35°C but had no effect on mycelial growth observed at 25°C. These results suggest that the viability of the fungus was not affected in the RNAi transformants and that the observed effects were due to the loss of thermotolerance. A yeast two-hybrid assay using SSCMK1 as bait revealed that this kinase interacts with SSHSP90 at the C terminal portion of HSP90. Inhibiting HSP90 brought about thermal intolerance in *S. schenckii *yeast cells and the development of a morphology at 35°C reminiscent of that observed in the SSCMK1 RNAi transformants. This suggests that the role of SSCMK1 in thermotolerance could be through its effects on SSHSP90. These results confirmed SSCMK1 as an important enzyme involved in the dimorphism of *S. schenckii*. This study constitutes the first report of the transformation of *S. schenckii *and the use of RNAi to study gene function in this fungus.

## Methods

### Strains

*S. schenckii *(ATCC 58251) was used for all experiments. Stock cultures were maintained in Sabouraud dextrose agar slants at 25°C as described previously [56]. *S. cerevisiae *strains AH109 and Y187 were used for the yeast two-hybrid screening and were supplied with the MATCHMAKER Two-Hybrid System (Clontech Laboratories Inc., Palo Alto, CA, USA).

### Culture conditions

*S. schenckii *yeast cells were obtained by inoculating conidia in 125 ml flask containing 50 ml of a modification of medium M. The cultures were incubated at 35°C with shaking at 100 rpm for 5 days as described previously [56]. Mycelia were obtained by inoculating conidia into a 125 ml flask containing 50 ml of this medium and incubated at 25°C without shaking. Solid cultures were obtained by inoculating conidia or yeast cells in a modification of medium M plates with added agar (15%) and/or geneticin (300 or 500 μg/ml) and incubated at 25°C or 35°C according to the experimental design.

For the growth determinations in the presence of geldanamycin (GdA, InvivoGen, San Diego, CA, USA), conidia from 10 day-old mycelial slants (10^9 ^cells/ml) were resuspended as described previously [56] and inoculated in 125 ml flasks containing 50 ml a modification of medium M with different concentrations of GdA (2, 5 and 10 μM). The cultures were incubated at 35°C with aeration and the growth recorded as OD 600 nm at 3, 5 and 7 days of incubation and compared to that of the controls containing only dimethyl sulfoxide (DMSO, 250 μl/50 ml of medium), the solvent used for resuspending GdA. The results were expressed as the OD at 600 nm of cells growing in the presence of geldanamycin/OD 600 nm of the controls ×100 ± one standard deviation of three independent determinations. The statistical significance of the differences observed in the data was analyzed using multiple comparisons with Student's T test and a Bonferroni correction was applied.

An aliquot of the cell suspension of the control cells and cells grown in geldanamycin (10 μM) containing medium were mounted on lactophenol cotton blue and observed microscopically after 7 days of incubation.

### Microscopy

Microscopic observations of the fungus were done using a Nikon Eclipse E600, equipped with a Nikon Digital Sight DS-2Mv and the NIS-Elements F 2.3 software from the Department of Pathology, Medical Sciences Campus, University of Puerto Rico.

### Nucleic acid isolation

DNA and total RNA from *S. schenckii *yeast cells was obtained as described previously [57]. Poly A^+ ^RNA was obtained from total RNA using the mRNA Purification Kit from Amersham Biosciences (Piscataway, NJ, USA) and used for the construction of the yeast two-hybrid library.

RNA for Real Time PCR (qRT-PCR) was obtained using the RiboPure™ Yeast rapid RNA isolation kit from Ambion Corp. (Austin, TX, USA). Briefly: up to 3 × 10**^8 ^**cells were collected by centrifugation and resuspended in lysis reagents (480 μl lysis buffer, 48 μl 10% SDS and 480 μl phenol:chloroform:IAA) the mixture was transferred to a tube containing cold zirconia beads and vortexed at a maximum speed for 10 min. The aqueous phase was transferred to a 15 ml conical tube followed by the addition of 1.9 ml of binding buffer and 1.25 ml of 100% ethanol and applied to a filter cartridge and centrifuged, 700 μl at a time. The RNA bound to the filter was washed once with wash solution 1 and twice with wash solution 2/3. The RNA was eluted with 50 μl of elution solution preheated at 95°C. The total RNA was treated with DNAse as described by the manufacturer. The concentration was determined using the NanoDrop**^® ^**ND-1000 UV-Vis Spectrophotometer (Thermo Fisher Scientific, Wilmington, DE, USA).

The RNA was transcribed to cDNA using the RETROscript**^® ^**Reverse Transcription kit (Ambion Inc.). Briefly: 2 μg of total RNA and 2 μl of Oligo (dT) were mixed and incubated for 3 min at 85°C. The remaining components were added in a stepwise manner: 2 μl of 10× RT Buffer, 4 μl dNTP mix, 1 μl RNase Inhibitor, 1 μl reverse transcriptase, and completed up to a final volume of 20 μl with water. The reaction was incubated at 44°C for 1 hr followed by 10 min at 92°C to inactivate the RT enzyme.

### Polymerase chain reaction (PCR) and Rapid amplification of cDNA ends (RACE)

For the identification of the Dicer-1 gene homologue in *S. schenckii*, degenerate primers were designed based on the sequence of conserved motifs in the *N. crassa *Dicer-1 gene (GenBank accession no. EAA32662) and modified according to the *S*. *schenckii *codon usage. PCR amplification was done using *S. schenckii *DNA as template and primers: Dicer-1 (fw) 5' tacatycagagccgsggscgsgcscgs 3' and Dicer-1 (rev) 5' gtcsagsaggctgtcsccsagraaytc 3'.

The Ready-to-Go™Beads (Amersham Biosciences) were used for PCR. All PCR reactions were carried out in the ABI PCR System 2720 (Applied Biosystems, Foster City, CA, USA). The PCR parameters used were: an initial denaturation step at 94°C for 1 min, followed by 30 cycles of denaturation at 94°C for 30 sec and extension at 72°C for 2 min. The annealing temperatures were adjusted according to the primers used. All PCR products obtained were analyzed using agarose gel electrophoresis and the DNA recovered using Spin-X Centrifuge Tube Filters as described by the manufacturer (0.22 μm, Corning Costar Corp., Corning, NJ, USA). The PCR products were cloned using the TOPO TA Cloning**^® ^**System (Invitrogen Corp., Carlsbad, CA, USA). The ligated PCR products were amplified by transformation of One Shot**^® ^***E.coli *Chemically Competent Cells. Plasmid preparations were obtained using the Fast Plasmid™Mini technology (Brinkmann Instruments, Inc. Westbury, NY, USA) as described by the manufacturer. Sequencing was done using Retrogen DNA Sequencing (San Diego, CA, USA).

*S. schenckii *cDNA was used as template for RLM-RACE (Applied Biosystems) to obtain additional sequence at the 5' end of the *S. schenckii sshsp90 *gene homologue as described by the manufacturer. All RACE reactions were carried out in the ABI PCR System 2720 (Applied Biosystems). The touchdown PCR and nested PCR parameters used for the initial RACE reactions were the same as described previously [57]. Nested primers were designed to improve the original amplification reactions. Bands from the 5' nested PCR were excised from the gel and cloned as described above. Primers for RACE were designed based on the sequence obtained from the yeast two-hybrid assay. For the 5' RACE of *sshsp90 *gene the following primers were used: AICRPRRL (rev) 5' aaagtcttcttggacgacatatagc 3' for the touchdown reaction and EKVVVSHKL (rev) 5' gtcagcttgtgggagacaacaacctt 3' and INVYSN (rev) 5' ttattggagtagacggtgttgat 3' for the nested reactions, DKDAKTLT (rev) 5' tcgtaagagtcttggcatccttgtc for the touchdown reaction and INTVYSN (rev) 5' tattggagtagacggtgttgat 3' for the nested reaction. For RT-PCR the following primers were used ISQLLSL (for) 5'atctctcagctcctgtctct 3' and FSAYLN (rev) 5'caaccaggtaagccgagtagaaa 3' and EQMDLY (for) 5'atgagcagatggactacctt 3' and YYITGES (rev) 5' gatggactcgccagtgatgtagtac. For PCR, DNA was used as template with primer ETFEFQ (for) 5' gagacgttygagttycaggc 3' and EKVVVSHKL as reverse primer. The RACE products were cloned as described above for PCR products, amplified and sequence using Davis Sequencing (Davis, CA, USA).

### RNAi plasmid and constructs

For RNAi experiments, pSilent-SD2G (pSD2G) developed by Nakayashiki and collaborators [32], and obtained from the Fungal Genetic Stock Center (FGSC) was used. This plasmid has a geneticin resistance cassette and two trpC promoters flanking the multiple cloning site (MCS) (Additional File 3). The pSD2G was amplified by transformation of One Shot**^® ^***E.coli *Chemically Competent Cells. Plasmid preparations were obtained using the Fast Plasmid™Mini technology (Brinkmann Instruments, Inc.) as described by the manufacturer.

Two different SSCMK1 PCR products were cloned in the multiple cloning site of pSD2G (Additional File 3A and 3B). For the construction of pSD2G-RNAi1, a 405 bp sequence of the 3' region of the *sscmk1 *gene (nucleotides 1194 to 1598) was amplified using *S. schenckii *cDNA as template and primers CaMK-RNAi1 (fw) 5' gctgaagcacaagtggct 3' and CaMK-RNAi1(rev) 5' ggtgagccctgcttgctg 3'. The conditions for amplification were: an initial denaturation step at 94°C for 1 min, followed by 30 cycles of denaturation at 94°C for 30 sec, annealing at 39°C for 1 min and extension at 72°C for 2 min. The PCR product was cloned in pCR^®^2.1-TOPO, sequenced and excised by digestion with EcoR1. The restriction product was cloned in the MCS of pSD2G to produce pSD2G-RNAi1 (Additional File 3A). For the construction of pSD2G-RNAi2, a 432 bp sequence of the 5' region of the *sscmk1 *gene (nucleotides 379 to 810) was amplified by PCR with primers: CaMKRNAi2 (fw) 5' atgagcttctctagtatg 3' and CAMKRNAi2 (rev) 5' ttttaggtctcgatgcac 3' using *S. schenckii *cDNA as template using the same conditions stated above. The cloned insert was sequenced and excised from the pCR^®^2.1-TOPO plasmid by digestion with XbaI and HindIII and cloned into pSD2G to produce pSD2G-RNAi2 (Additional File 3B).

Cloning of the inserts into the linearized plasmid was performed using the Quick T4 DNA Ligase (New England Biolabs, Ipswich, MA, USA) as described by the manufacturer. Plasmid preparations were obtained using the Qiagen Plasmid Midi kit (Qiagen Corp., Valencia, CA, USA), as described by the manufacturer. Confirmation of the inserted sequence was done using the Retrogen DNA Sequencing.

### Transformation

The transformation protocol used was a modification of the method described for *Ophiostoma *[33]. Briefly: yeast cells (approximately 10^9 ^cells) were collected by centrifugation, washed with sterile distilled water, resuspended in 50 ml of Solution A (25 mM β-mercaptoethanol, 5 mM Na_2_EDTA, pH 8.0) and incubated for 20 min at 25°C with gentle shaking. The cells were centrifuged and re-suspended in 1 M MgSO_4_, re-centrifuged and incubated in 10 ml (10 mg/ml) of Glucanex**^®^**(Sigma-Aldrich, St. Louis, MO, USA) for 2 hours at 25°C with gentle agitation. Forty ml of STC (1 M sorbitol, 25 mM Tris HCl, 50 mM CaCl_2_) solution were added and the cell suspension centrifuged. The pellet was resuspended in 6 ml of STC and 3 aliquots of 200 μl each of the protoplast suspension were transferred to 50 ml centrifuge tubes. The following compounds were added in a stepwise manner: 1 μl of β-mercaptoethanol, 10 μg of transforming DNA (pSD2G-RNAi1 or 2, or pSD2G), 50 μl of a 66% PEG 3,350 solution in 25 mM CaCl_2_/25 mM Tris-HCl and 10 μl of denatured salmon sperm DNA (10 mg/ml). After a 20 minutes incubation at 25°C, an additional 2.5 ml of PEG solution was added in aliquots of 1 drop, 0.5 ml and 2 ml, and incubated for 20 minutes at 25°C. One, five and thirty ml of STC were added to the protoplast suspension. The suspension was centrifuged for 20 min at 1,500 rpm (450 × g) and the pellet resuspended in 1 ml of a modification of medium M (1 M sorbitol). After a recovery period of 3 hours at 35°C with gentle agitation, 200 μl aliquots were plated on geneticin (300 μg/ml) containing medium M agar plates and incubated at 35°C until colonies appear (7-10 days). For RNAi controls, cells were transformed with pSD2G. Further transfers of colonies were done in medium M agar plates containing geneticin (500 μg/ml) and the growth resuspended in this same medium without agar and stored at -80°C for further studies.

### Colony PCR of transformants

For colony PCR, growth from the colonies obtained after transformation were resuspended in sterile PCR water and used as template for PCR. Colony PCR of transformants was used to corroborate the presence of the plasmid pSilent-Dual2G in the transformed colonies. The primers used for the determination of the presence of the transforming plasmids were: G418 (fw) 5' ctgaatgaactgcaggacga 3' and G418 (rev) 5' agaactcgtcaagaaggcga 3'. These primers amplify a 622 bp fragment of the geneticin resistance cassette. The PCR parameters were as follows: an initial denaturation step at 94°C for 2 min, followed by 35 cycles of denaturation step at 94°C for 1 min, annealing at 45°C for 1 min, and extension at 72°C for 2 min. PCR products were analyzed on agarose gels for the presence of a band of the expected size.

### Real-Time PCR

The *sscmk1 *gene cDNA cloned in pCR^®^2.1-TOPO plasmid in *E.coli *Top10 cells was obtained from the cDNA collection of the laboratory and was used as template for Real Time PCR standard curve. The coding region of the *sscmk1 *gene was amplified using the insert containing plasmid as template and primers MSFSSM-CMK (fw) 5'atgagcttctctagtatg 3' and KQGSP-CMK (rev) 5' tcaaggtgagccctgctt 3'. The PCR product was excised from the gel using Spin-X Centrifuge Tube Filters as described by the manufacturer (0.22 μm, Corning Costar Corp.) and the concentration of DNA quantified using the NanoDrop**^® ^**ND-1000 UV-Vis Spectrophotometer (Thermo Fisher Scientific). Different dilutions of this cDNA were used as template for the amplification of a short region of 86 bp from the *sscmk1 *gene comprised between nucleotides 632-717. The primers were: SSCMK1 (fw) 5'ggtttgaatcgagggata 3' and SSCMK1 (rev) 5' cttgccctgctcacaaat 3'. PCR was performed with iQ™ SYBR^® ^Green Supermix (Bio-Rad Laboratories, Hercules, CA, USA) using a primer concentration of 400 nM and 5 μl of the cDNA dilution (10-100 ng of cDNA) as a template in a total volume of 25 μl. Reactions were set up with 2 replicates per sample. Controls without templates were included for the primer set. PCR cycling parameters were 95°C for 3 min, then 50 cycles at 95°C for 10 sec and 57°C for 1 min (data collection and real time analysis enabled) followed by 1 min at 95°C, 1 min at 55°C and 100 cycles at 55°C for 10 sec increasing temperature after cycle 2 by 0.4°C (melting curve data collection and analysis enabled). Fluorescence emissions were detected with using the iCycler Real-Time PCR Detection System (Bio-Rad Laboratories). A standard curve was constructed of log of ng of *sscmk1 *cDNA *vs *Ct.

The RNA was extracted from cells transformed with pSD2G and cells transformed with pSD2G-RNAi1 and converted to cDNA as described above. The same primers used for the standard curve were used for the samples. Cells transformed with pSD2G-RNAi1 or pSD2G were grown in 50 ml of a modification of medium M with 500 μg/ml geneticin at 35°C and cell growing in plates of medium M with 500 μg/ml geneticin and 15% agar at 25°C according to the experimental design. RNA was extracted as mentioned above and converted to cDNA using the RETROscript^® ^First-Strand Synthesis Kit (Ambion Inc.). The levels of *sscmk1 *RNA in cells transformed with pSD2G-RNAi1 and pSD2G was determined using the iCycler Real-Time PCR Detection System (Bio-Rad Laboratories) as described above. The same 86 bp region mentioned above was amplified using *S. schenckii *cDNA from transformed cells as template and the same primers mentioned above. Each 25 μl reaction consisted of 20 μl of a master mix (1× SYBR Green SuperMix, 400 nM of each primer) and 5 μl of cDNA. Real-Time PCR amplification parameters were: an initial denaturation step at 95°C for 3 min, then 50 cycles at 95°C for 10 sec and 57°C for 1 min (data collection and real time analysis enabled) followed by 1 min at 95°C, 1 min at 55°C and 100 cycles at 55°C for 10 sec increasing temperature after cycle 2 by 0.4°C (melting curve data collection and analysis enabled). A minimum of 3 independent experiments were performed for each transformant. The average ± the standard deviation of the ng of *sscmk1 *RNA/ng of total RNA was calculated using the standard curve. The Student's T test was used to determine the significance of the data (p < 0.05).

### Yeast two-hybrid assay

MATCHMAKER Two-Hybrid System was used for the yeast two-hybrid assay using 3 different reporter genes for the confirmation of truly interacting proteins (Clontech Laboratories Inc.) as described previously by us [58]. For the construction of the SSCMK1 bait plasmid, a pCR^®^2.1-TOPO plasmid (Invitrogen Corp.) containing the *sscmk1 *gene cDNA sequence of *S. schenckii *from the laboratory collection was used as template for PCR to obtain the coding sequence of the gene. *E. coli *TOP10 One Shot^® ^chemically competent cells (Invitrogen Corp.) containing the plasmid were grown in 3 ml of LB broth with kanamycin (50 μg/ml) at 37°C for 12 to 16 hours and the plasmid isolated with the Fast Plasmid™ Mini Kit (Brinkmann Instruments, Inc.). The *sscmk1 *insert was amplified by PCR using Ready-to-Go™Beads (Amersham Biosciences) and primers containing the gene sequence and additional sequences containing restriction enzyme sites for EcoR1 and XmaI added at the 5' and 3'ends. The primers used were: SSCMK1-Eco (fw) 5' taccggaattccccatgagcttctct 3' and SSCMK1-Xma (rev) 5' cccgggtcaaggtgagccctgcttg 3'. The *sscmk1 *cDNA sequence with the added restriction enzyme site was cloned in the same vector, amplified and purified using the QIAfilter Plasmid Purification kit (Qiagen Corp.). The *sscmk1 *gene was excised from the vector by enzymatic digestion with EcoR1 and XmaI. The pGBKT7 plasmid vector was linearized using the same enzymes mentioned above. The restriction digested *sscmk1 *gene and the linearized pGBKT7 were ligated using the Quick Ligation™ Kit (New England Biolabs, Inc.). The ligation reaction was incubated at 25°C for 5 min, chilled on ice, and used to transform *E. coli *TOP10 One Shot^® ^chemically competent cells. The correct orientation and frame of the inserted gene sequence was verified by sequencing. The bait containing plasmid was isolated using Fast Plasmid™Mini technology (Brinkmann Instruments) and used to transform competent *S. cerevisiae *yeast cells (Y187) with the YEAST- MAKER™ Yeast Transformation System 2 (Clontech Laboratories Inc.). Tests for autonomous gene activation and cell toxicity were carried out as described by the manufacturer.

A cDNA library using *S. schenckii *yeast RNA was constructed as described previously in AH109 cells [58]. Transformants were selected in SD/-Leu plates, harvested and used for mating with the bait containing *S. cerevisiae *strain Y187. Mating of *S. cerevisiae *yeast cells strains Y187 (Mat-α) and AH109 (Mat-a) was done according to the manufacturer's instructions as described previously. Colonies growing in triple dropout medium (TDO) SD/-Ade/-Leu/-Trp were tested for growth in quadruple dropout medium (QDO) SD/-Ade/-His/-Leu/-Trp. These positive colonies were re-plated in QDO medium to verify that they maintained the correct phenotype.

Colony PCR was used to corroborate the presence of both plasmids in the diploid cells using the T7/3'BD sequencing primer pair for the pGBKT7/SSCMK1 plasmid and the T7/3'AD primer pair for the pGADT7-Rec library plasmid and yeast colony suspension as template. The Ready-to-Go™ Beads (Amersham Biosciences) were used for PCR. The amplification parameters were those described previously [58]. PCR products were analyzed on agarose gels and the DNA recovered using Spin-X Centrifuge Tube Filters as described by the manufacturer (0.22 μm, Corning Costar Corp.). The PCR products were cloned and amplified as described previously [58]. Plasmid preparations were obtained using the Fast Plasmid™ Mini technology (Brinkmann Instruments) and the inserts sequenced using commercial sequencing services from SeqWright (Fisher Scientific, Houston, TX, USA) and Retrogen DNA Sequencing (Retrogen Inc., San Diego, CA, USA)).

### Co-immunoprecipitation (Co-IP) and Western blots

Co-immunoprecipitation followed by Western blot was used to confirm the interaction of HSP90 identified in the yeast two-hybrid analysis as interacting with SSCMK1 as described previously [58]. *S. cerevisiae *diploids obtained in the yeast two-hybrid assay were grown in QDO, harvested by centrifugation and resuspended in 8 ml containing phosphate buffer saline (800 μl) with phosphatase (400 μl), deacetylase (80 μl) and protease inhibitors (50 μl), and PMSF (50 μl). The cells were broken as described previously [59]. The cell extract was centrifuged and the supernatant used for Co-IP using the Immunoprecipitation Starter Pack (GE Healthcare, Bio-Sciences AB, Bjorkgatan, Sweden). Briefly, 500 μl of the cell extract (1-2 μg of protein/ml) were combined with 1-5 μg of the anti-cMyc antibody (Clontech Laboratories Inc.) and incubated at 4°C for 4 h, followed by the addition of protein G beads and incubated at 4°C overnight in a rotary shaker. The suspension was centrifuged and the supernatant discarded, 500 μl of the wash buffer added followed by re-centrifugation. This was repeated 4 times. The pellet was resuspended in Laemmeli buffer (20 μl) with β-mercaptoethanol (5%) and heated for 5 min at 95°C, centrifuged and the supernatant used for 10% SDS PAGE at 110 V/1 h. Pre-stained molecular weight markers (BioRad, Corp.) were run in the gel.

Electrophoretically separated proteins were transferred to nitrocellulose membranes using the BioRad Trans Blot System^® ^for 1 h at 20 volts and blocked with 3% gelatin in TTBS (20 mM Tris, 500 mM NaCl, 0.05% Tween-20, pH 7.5) at room temperature for 30-60 min. The strips were washed with TTBS and incubated overnight in the antibody solution containing 20 μg of antibody, anti-cMyc or anti-HA (Clontech Laboratories Inc.). Controls where the primary antibody was not added were included. The antigen-antibody reaction was detected using the Immun-Star™AP Chemiluminescent protein detection system from BioRad Corporation as described by the manufacturer in a BioRad Versa-Doc Gel Imaging System (BioRad, Corp).

### Bioinformatics Sequence Analysis

The theoretical molecular weights of the proteins were calculated using the on-line ExPASy tool http://expasy.org/tools/pi_tool.html. On-line NCBI Conserved Domains Database http://www.ncbi.nlm.nih.gov/cdd [60] and Pfam http://pfam.sanger.ac.uk/search [61] searches were used to identify potential motifs present in SSDCL-1 and SSHSP90. The protein classification was performed using the PANTHER Gene and Protein Classification System http://www.PANTHERdb.org [62]. On-line database searches and comparisons for SSDCL-1 and SSHSP90 were performed with Integrated Protein Classification (iProClass) database http://pir.georgetown.edu/pirwww/dbinfo/iproclass.shtml [63] and the BLAST algorithm http://www.ncbi.nlm.nih.gov/BLAST/ with a cutoff of 10^-7^, a low complexity filter and the BLOSUM 62 matrix [64]. Multiple sequence alignments were built using M-COFFEE http://www.igs.cnrs-mrs.fr/Tcoffee/tcoffee_cgi/index.cgi::Regular [65,66]. The alignments were visualized using the program GeneDoc http://www.psc.edu/biomed/genedoc. The GenBank accession numbers for the multiple sequence alignment for SSDCL-1 homologues were: *Chaetomium globosum*, XP_001223948.1; *Podospora anserina*, XP_00190115.1; *N.crassa*, XP_961898; *Magnaporthea grisea*, A4RKC3.2; *Cryphonectria parasitica*, Q2VF19.1; *Sclerotinia sclerotiorum*, XP_001585179.1 and *Gibberella zeae*, XP_389201.1. The GenBank accession numbers for the multiple sequence alignment for SSHSP90 homologues were: *P. brasiliensis*, AAX33296.1; *P.anserina*, XP_0019911127.1; *A. nidulans*, XP_681538.1; *Ajellomyces dermatitidis*, XP_002624667.1; *Phaeosphaeria nodorum*, XP_001791544.1; *S. cerevisiae*, EGA76545.1; *Grosmannia clavigera*, EFX01463.1; *Homo sapiens *(alpha isoform 2), NP_005339.

## Competing interests

The authors declare that they have no competing interests.

## Authors' contributions

JRC did the transformation, RNAi experiments and the yeast two-hybrid assay that identified HSP90 a protein that interacts with SSCMK1. JRC also did the Co-IP experiments and the partial sequencing of SSDCL-1 and SSHSP90. This work was done as part of his research for the PhD. degree. The library used for the yeast two-hybrid assay was done by WGV. She also participated in the sequencing of SSHSP90. LPS participated in the bioinformatics study of the SSDCL-1 and participated in the sequencing and bioinformatics analysis of SSHSP90. RGM participated and supervised the bioinformatics study of the proteins and data calculations. NRV designed the study, drafted the manuscript, participated in sequence alignments, data and statistical calculations, and domain characterizations. All authors have read and approved the final manuscript.

## Supplementary Material

Additional file 1 **DNA and Amino acid sequence SSDCL-1**. The partial DNA and derived amino acid sequence of the *ssdcl-1 *gene. Non-coding regions are given in lower case letters, coding regions and amino acids are given in upper case letters. The helicase domain is shadowed in yellow, the dsRNA binding domain is shadowed in blue green and the RNAse 3 domain is shadowed in gray. The putative intron is given in lower case red letters. Click here for file

Additional file 2 **Amino acid sequence alignments of SSDCL-1 to other fungal DCL-1 homologues**. The predicted amino acid sequence of *S. schenckii *SSDCL-1 and DCL-1 homologues from other fungi were aligned using M-Coffee. In the alignment, black shading with white letters indicates 100% identity, gray shading with white letters indicates 75-99% identity, gray shading with black letters indicates 50-74% identity. Important domains are highlighted in colored boxes. The helicase domain, dsRNA binding domain and the RNAse III domains are highlighted in green, red and blue boxes, respectively. Click here for file

Additional File 3 **pSD2G, *sscmk1 *inserts and colony PCR**. This file shows pSD2G (pSD2G) from the Fungal Genetic Stock Center. It has a geneticin resistance cassette and two trpC promoters flanking the multiple cloning site (MCS). File 3A and 3B show the nucleotide sequences of the *sscmk1 *gene inserted into pSD2G: a 405 bp insert from the 3' region and a 432 bp insert from the 5' region of the gene. These inserts were amplified by PCR from cDNA containing the coding sequence of the *sscmk1 *gene, cloned in pCR**^®^**2.1-TOPO, excised by digestion with restriction enzymes and cloned in the MCS of pSD2G to produce pSD2G-RNAi1 and pSD2G-RNAi2, respectively. File 3C Shows the results of the colony PCR of various *S. schenckii *transformants. Cell suspensions of *S. schenckii *transformants were used as templates for PCR using the G418 (for)/G418 (rev) primer pair as described in Methods. Lane 4 shows the 123 bp DNA ladder. Lanes 1, 2, 3, 5 and 6 shows the bands obtained when the cells transformed with pSD2G-RNAi1 from colonies 14, 15, 18, 19 and 21 were used as template, respectively. In lanes 7 and 8, suspensions of non-transformed cells were used as templates for PCR. A band of the expected size, 622 bp, due to the presence of the geneticin resistance cassette was observed in transformed yeast cells. Click here for file

Additional File 4 **cDNA and derived amino acid sequence of the *S. schenckii *HSP90 homologue isolated using yeast two-hybrid assay**. The cDNA and derived amino acid sequence of the SSHSP90 identified in the yeast two-hybrid assay as interacting with SSCMK1 is shown. Non-coding regions are given in lower case letters, coding regions and amino acids are given in upper case letters. The HATPase domain is shaded in yellow and the sequence isolated in the yeast two-hybrid assay is shaded in gray. Red letters mark the conserved MEEVD domain in the C terminal domain of HSP90, necessary for the interaction with tetratricopeptide repeat containing proteins. Click here for file

Additional File 5 **Amino acid sequence alignment of SSHSP90 to other fungal HSP90 homologues**. The predicted amino acid sequence of *S. schenckii *SSHSP90 and HSP90 homologues from other fungi were aligned using M-Coffee. In the alignment, black shading with white letters indicates 100% identity, gray shading with white letters indicates 75-99% identity, gray shading with black letters indicates 50-74% identity. Important domains, the HATPase domain and theHSP 90 domain, are highlighted in blue and red boxes, respectively. The C terminal domain is indicated with a blue line. Click here for file
