# Supplementary material for: Calcium/calmodulin kinase1 and its relation to thermotolerance and HSP90 in Sporothrix schenckii: an RNAi and yeast two-hybrid study
Source: BMC Microbiol. 2011 Jul 11;11:162. doi: 10.1186/1471-2180-11-162 (PMC3146815; doi:10.1186/1471-2180-11-162)
Supplement: Additional file 1 — DNA and Amino acid sequence SSDCL-1. The partial DNA and derived amino acid sequence of the ssdcl-1 gene. Non-coding regions are given in lower case letters, coding regions and amino acids are given in upper case letters. The helicase domain is shadowed in yellow, the dsRNA binding domain is shadowed in blue green and the RNAse 3 domain is shadowed in gray. The putative intron is given in lower case red letters. [file 1471-2180-11-162-S1.PDF]

```
   1 - ATAACAATCTGCTGCACGAAATTTCCAAGCCCAAGAAGGAGCGCATCGTGCACTACCACACGGCCCTGGTCCAACACGAGT - 81
   1 -  N  N  L  L  H  E  I  S  K  P  K  K  E  R  I  V  H  Y  H  T  A  L  V  Q  H  E  S - 27
  82 - CAAACCTCATGCGGCGGCTCCGGCAGCTCGTGGGCAACCACAAGCTCTTTGCTCGTAACTTCCAGTACGCGGACGCCAACC - 162
  28 -  N  L  M  R  R  L  R  Q  L  V  G  N  H  K  L  F  A  R  N  F  Q  Y  A  D  A  N  L - 54
 163 - TCTCGGTGCTGGGGCCTTGGATCATCGACCGCTTCTGGCAGATCCCGTTCCGCACCGAGGAACTCGCCAAACAGGAGGCGA - 243
  55 -  S  V  L  G  P  W  I  I  D  R  F  W  Q  I  P  F  R  T  E  E  L  A  K  Q  E  A  K - 81
 244 - AGGCCGAGATTGAGAGACAGCCCGACGACGAGGGTGCGGGACGGAACCCCCTCAAGCTCGACGGTCTCGGCGACGAGCCCG - 324
  82 -  A  E  I  E  R  Q  P  D  D  E  G  A  G  R  N  P  L  K  L  D  G  L  G  D  E  P  V - 108
 325 - TGCAGTACAACTCTAATGTGGTAGCTGTGCGGGACGCCAGTCGTTTCGTGGAGCTGTACCGCTTCGTGCCGCCGACTATGG - 405
 109 -  Q  Y  N  S  N  V  V  A  V  R  D  A  S  R  F  V  E  L  Y  R  F  V  P  P  T  M  A - 135
 406 - CCCAGTTGTCGAACAAAGTTCAGCGGCTGCACGACGCCCTTTTCGACGTGTTCACGCGGTTCAGCGGCCAGAAGACGCGGT - 486
 136 -  Q  L  S  N  K  V  Q  R  L  H  D  A  L  F  D  V  F  T  R  F  S  G  Q  K  T  R  C - 162
 487 - GCATCGTCTTTGTAGAGCAGCGCTACACAGCCACCCTTTTGGCCGACCTGTTCCAGCAGGAGCAAATGAAGATCCCAAACC - 567
 163 -  I  V  F  V  E  Q  R  Y  T  A  T  L  L  A  D  L  F  Q  Q  E  Q  M  K  I  P  N  L - 189
 568 - TTCGGACCGGCGTCTTGGTCGGCGGCGGATCCAAAGACATGGGCAAGAACACGTTCCGCACGCAGCTGCTGACCATCTCCA - 648
 190 -  R  T  G  V  L  V  G  G  G  S  K  D  M  G  K  N  T  F  R  T  Q  L  L  T  I  S  K - 216
 649 - AGTTCAAGCGTGGCCAGGTCAACTGCCTCTTCGCGACATCCATTGCTGAGGAGGGCCTGGACATTCCGGGACTGCAACCTT - 729
 217 -  F  K  R  G  Q  V  N  C  L  F  A  T  S  I  A  E  E  G  L  D  I  P  G  L  Q  P  C - 243
 730 - GTCATCCGCTTCGACTATACGGCACCATGATCCAGTATATTCAGTCGCGCGGGCGAGCCCGGCACCAGGAGTCGACATACA - 810
 244 -  H  P  L  R  L  Y  G  T  M  I  Q  Y  I  Q  S  R  G  R  A  R  H  Q  E  S  T  Y  I - 270
 811 - TCCACATGGCCGAGCTCGGCAACGTGGATCACCGCCGCCTGCTCGCGGAGAATAAGGCCAGCGAGAACAAGATGCGCGACT - 891
 271 -  H  M  A  E  L  G  N  V  D  H  R  R  L  L  A  E  N  K  A  S  E  N  K  M  R  D  F - 297
 892 - TTTGCAACGCGCTGCCGGAAAACCGCAAGCTGGAGGGAAATGATAATGACATGGACTACTTTTTGCGCGACGAGACAGACC - 972
 298 -  C  N  A  L  P  E  N  R  K  L  E  G  N  D  N  D  M  D  Y  F  L  R  D  E  T  D  Q - 324
 973 - AGCAAGTGTATGTTGTGCCGTCCACCCAGGCGAAGCTGACGTACCGGTCCAGTCTGGTCATCCTGGCCCAGTATGTGTCGA - 1053
 325 -  Q  V  Y  V  V  P  S  T  Q  A  K  L  T  Y  R  S  S  L  V  I  L  A  Q  Y  V  S  T - 351
1054 - CGCTCCCCGAGCCGAGCGAGGGAGTGCCGAAGCCCGAGTACTCTGTGTTTTGCACCGCCGACGGCTTCGTCTGCGAGGTGG - 1134
 352 -  L  P  E  P  S  E  G  V  P  K  P  E  Y  S  V  F  C  T  A  D  G  F  V  C  E  V  V - 378
1135 - TGTTGCCATCGTCTTCGCCCATCCGCCAGGCGACCGGCCGACCCCATAGCCGGAAGCAGGTCGCCAAGTGCGCGGCGGCCT - 1215
 379 -  L  P  S  S  S  P  I  R  Q  A  T  G  R  P  H  S  R  K  Q  V  A  K  C  A  A  A  F - 405
1216 - TTGCCATGTGCCTCAAACTGTACGAAAAGAAGTACATTGACAAGCATCTGCACCCCATCTTTGCGTCCCGGCTGCCTGCCA - 1296
 406 -  A  M  C  L  K  L  Y  E  K  K  Y  I  D  K  H  L  H  P  I  F  A  S  R  L  P  A  M - 432
1297 - TGCGCAACGCGCGGCTGGCCGTCAGCTCCAAGAAGCAGGCCCAGTACACGATGCGCGTCAAGCCCGAGACATGGTCGGTCC - 1377
 433 -  R  N  A  R  L  A  V  S  S  K  K  Q  A  Q  Y  T  M  R  V  K  P  E  T  W  S  V  L - 459
1378 - TCGGCATGCCGACACAGCTGTATGCTTCGATTCTCTCGCTCACCCAACCGGAGTCCCTGGACCGTCCCTCGCGCCCGCTCG - 1458
 460 -  G  M  P  T  Q  L  Y  A  S  I  L  S  L  T  Q  P  E  S  L  D  R  P  S  R  P  L  V - 486
1459 - TGTTCTTGTCTCGGCACAAGATGCCTCTGCTCCCCGAGTTCCCCTTGTTCTTTAGCAAAGGCCGGGCCTCGAAGGTCCGCT - 1539
 487 -  F  L  S  R  H  K  M  P  L  L  P  E  F  P  L  F  F  S  K  G  R  A  S  K  V  R  C - 513
1540 - GCGTCCCCGTGGACATTCCCATGCAGCCTACCCCGAACGAGCTGGAGGCGCTCGCTGCGTATACCCTTCGCGCATTCTATG - 1620
 514 -  V  P  V  D  I  P  M  Q  P  T  P  N  E  L  E  A  L  A  A  Y  T  L  R  A  F  Y  D - 540
1621 - ATGTTTTCAGCAAGGAATACGAGGGCACGTCTAAGGACATGCCGTATTTCATTGCCCCGTACAGCGAGCCCACGCACGACG - 1701
 541 -  V  F  S  K  E  Y  E  G  T  S  K  D  M  P  Y  F  I  A  P  Y  S  E  P  T  H  D  V - 567
1702 - TCGTGCTGCCGGACAAGAGCACTTGCCCTGATGCACGCTCGATCTTGGACTGGACTAGCATCCACCACGTCGCCGCACAGA - 1782
 568 -  V  L  P  D  K  S  T  C  P  D  A  R  S  I  L  D  W  T  S  I  H  H  V  A  A  Q  I - 594
1783 - TTGAGAGGATTACGTGCCAGGGCGACGAGCCCGATTCGTTTTTTGAAAACAAGTTTGTGTCGGACCCCTTTGACGGCTCGC - 1863
 595 -  E  R  I  T  C  Q  G  D  E  P  D  S  F  F  E  N  K  F  V  S  D  P  F  D  G  S  R - 621
1864 - GCAAGTTCTATTTGCGGCAGGTGCGGCGCGATATGTCGGCGCTCGATCCGGTGCCCGAGGGTGCGCCGGCGCCAAAGCACC - 1944
 622 -  K  F  Y  L  R  Q  V  R  R  D  M  S  A  L  D  P  V  P  E  G  A  P  A  P  K  H  R - 648
1945 - GTGCTTGGAACCGGCCGGACACGGTGCGCAACATTTTGAACTACAGCGTGAGCCTGTGGTCCAAGTCGCGGGTCAACACGC - 2025
 649 -  A  W  N  R  P  D  T  V  R  N  I  L  N  Y  S  V  S  L  W  S  K  S  R  V  N  T  P - 675
2026 - CGATCCGGCAAGACCAGGTTGTTGTGGAGGCGGATATCGTATACCAGCGCCGCAACCTGCTCGACGAGCGCGTGTTTCTCG - 2106
 676 -  I  R  Q  D  Q  V  V  V  E  A  D  I  V  Y  Q  R  R  N  L  L  D  E  R  V  F  L  D - 702
2107 - ACGATTTTGGACCTAGCCGGTGCTTTATTGTCTTGGATACACTTCGTATTTCGCCAgtatgtcattcccgccgcccctggc - 2187
 703 -  D  F  G  P  S  R  C  F  I  V  L  D  T  L  R  I  S  P                            - 720
2188 - tggtccgtgtaagttggctttttgtcgttgtgcttgccctgactctgtagATCCCTGTTGATTCGATTGCCATGATTTTCA - 2268
 721 -                                                    I  P  V  D  S  I  A  M  I  F  N - 731
2269 - ATTTGCCGCCCATCATCCACCGCATCGAGTCGACGCTCATTGCGTTGGAGGCCGCGGCCGTGATTGGGTTGCCTAGTATCT - 2349
 732 -  L  P  P  I  I  H  R  I  E  S  T  L  I  A  L  E  A  A  A  V  I  G  L  P  S  I  Y - 758
2350 - ACCCCGGCCTCGCACTGGAAGCGTGCACCAAAGACTGTGACGTTTCCGACGACGCGGGCGCGACCGAGACCGCCATTAACT - 2430
 759 -  P  G  L  A  L  E  A  C  T  K  D  C  D  V  S  D  D  A  G  A  T  E  T  A  I  N  F - 785
2431 - TCCAGGCCGGCATGGGCCGCAACTATGAACGACTCGAACTGTTGGGCGACTCGTTCCTAAAAATGGCCTCTACGATTGCAC - 2511
 786 -  Q  A  G  M  G  R  N  Y  E  R  L  E  L  L  G  D  S  F  L  K  M  A  S  T  I  A  L - 812
2512 - TCTACACGCTCGCCCCGGACAAGAATGAGTTTGAATACCACGTGGAGCGGATGTGCATGATCTGCAATAAGAACTTGTTCA - 2592
 813 -  Y  T  L  A  P  D  K  N  E  F  E  Y  H  V  E  R  M  C  M  I  C  N  K  N  L  F  N - 839
2593 - ACAATGCGCTCGAGATAGGGCTTGAGGAGTACATCCGCTCCAAAGAGTTTGACCGTGCTTGGTACCCGCCCGTTGTCGATG - 2673
 840 -  N  A  L  E  I  G  L  E  E  Y  I  R  S  K  E  F  D  R  A  W  Y  P  P  V  V  D  E - 866
2674 - AGACAGTGAAGTTGGCAGCAGAGGCGGCGGCCAAGACTGGCACACCCGACGAAAACGGCAAAACACCCACCGCACTGCACG - 2754
 867 -  T  V  K  L  A  A  E  A  A  A  K  T  G  T  P  D  E  N  G  K  T  P  T  A  L  H  G - 893
2755 - GCTTGATTCTCAAGAAAGGAAGAAAGCAGCAAGAGGGTAGCTACCATGCGCTGAGTGACAAGAGCATTGCCGATGTGTGCG - 2835
 894 -  L  I  L  K  K  G  R  K  Q  Q  E  G  S  Y  H  A  L  S  D  K  S  I  A  D  V  C  E - 920
2836 - AGGCCATGATTGGCGCCGCCTACCTCACCACATACGAGGAGCGCGACTTTGATTTGGCCGTCCAGGCTGTGTCTGTTGTGG - 2916
 921 -  A  M  I  G  A  A  Y  L  T  T  Y  E  E  R  D  F  D  L  A  V  Q  A  V  S  V  V  A - 947
2917 - CCAAGAGCAAGTTCCACCCCATGAGAACCTACAAGGAGTACTTTGCAGCCTACGTCCTGCCGGAGTGGCAGATCGCGCCCG - 2997
 948 -  K  S  K  F  H  P  M  R  T  Y  K  E  Y  F  A  A  Y  V  L  P  E  W  Q  I  A  P  A - 974
2998 - CCACCGCCGCCCAGGTGCATATGGCCAAAGCCGTGGCGCGTGACGTTGGTTACACCTTTACGTACCCGCGCCTGCTGCGCT - 3078
 975 -  T  A  A  Q  V  H  M  A  K  A  V  A  R  D  V  G  Y  T  F  T  Y  P  R  L  L  R  C - 1001
3079 - GCGCCGTCATGCATCCGTCGTACCCGCGCGTGTACGAGCAGCTGCCGAGCTACCAGCGCCTC                   - 3140
1002 -  A  V  M  H  P  S  Y  P  R  V  Y  E  Q  L  P  S  Y  Q  R  L  -                   - 1021
```
