# Supplementary material for: Calcium/calmodulin kinase1 and its relation to thermotolerance and HSP90 in Sporothrix schenckii: an RNAi and yeast two-hybrid study
Source: BMC Microbiol. 2011 Jul 11;11:162. doi: 10.1186/1471-2180-11-162 (PMC3146815; doi:10.1186/1471-2180-11-162)
Supplement: Additional file 2 — Amino acid sequence alignments of SSDCL-1 to other fungal DCL-1 homologues. The predicted amino acid sequence of S. schenckii SSDCL-1 and DCL-1 homologues from other fungi were aligned using M-Coffee. In the alignment, black shading with white letters indicates 100% identity, gray shading with white letters indicates 75-99% identity, gray shading with black letters indicates 50-74% identity. Important domains are highlighted in colored boxes. The helicase domain, dsRNA binding domain and the RNAse III domains are highlighted in green, red and blue boxes, respectively. [file 1471-2180-11-162-S2.PDF]

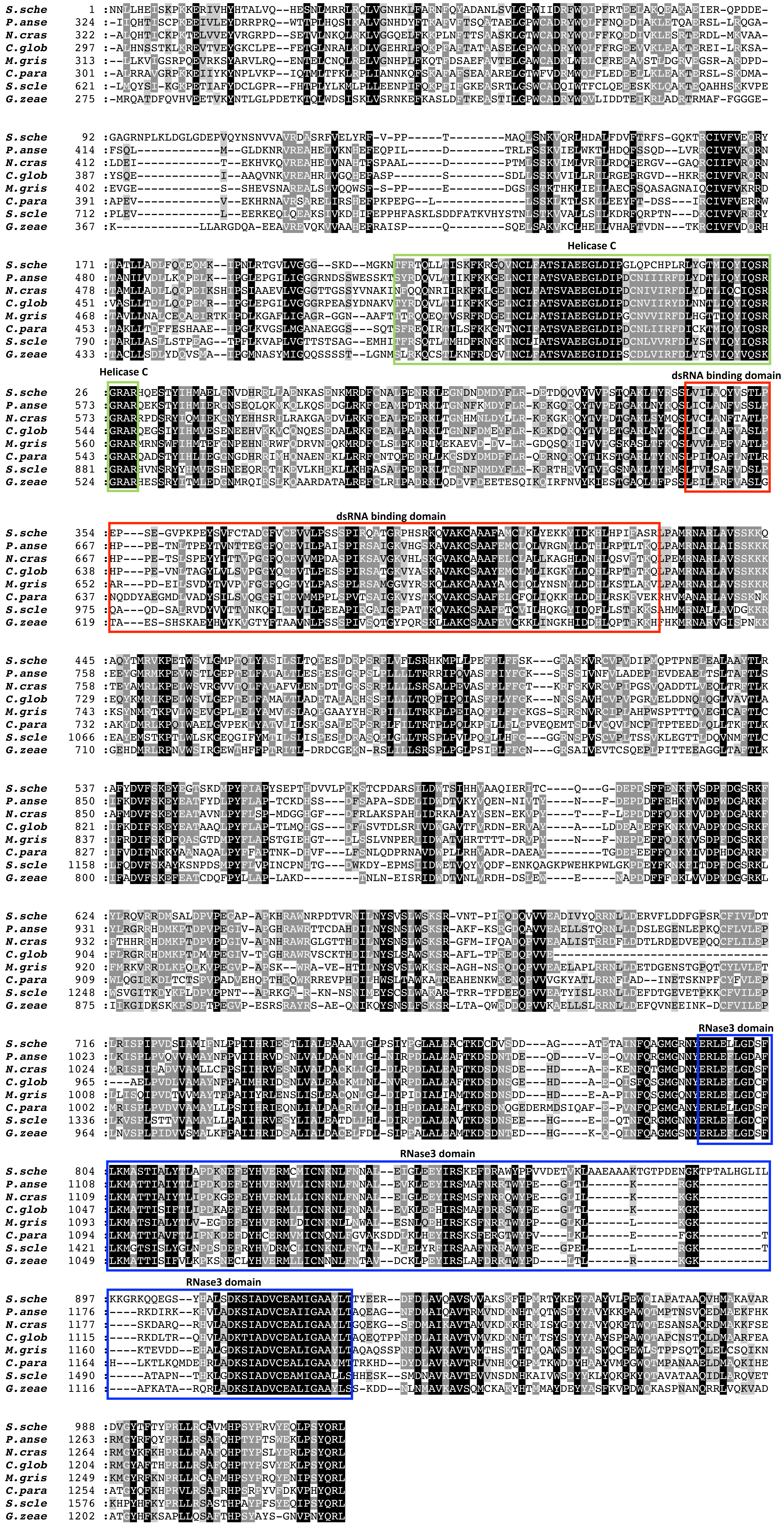
Helicase C
Helicase C
dsRNA binding domain
dsRNA binding domain
RNase3 domain
RNase3 domain
RNase3 domain
S.sche
P.anse
N.cras
C.glob
M.gris
C.para
S.scle
G.zeae
