# Supplementary figures and images for: Calcium/calmodulin kinase1 and its relation to thermotolerance and HSP90 in Sporothrix schenckii: an RNAi and yeast two-hybrid study
Source: BMC Microbiol. 2011 Jul 11;11:162. doi: 10.1186/1471-2180-11-162 (PMC3146815; doi:10.1186/1471-2180-11-162)

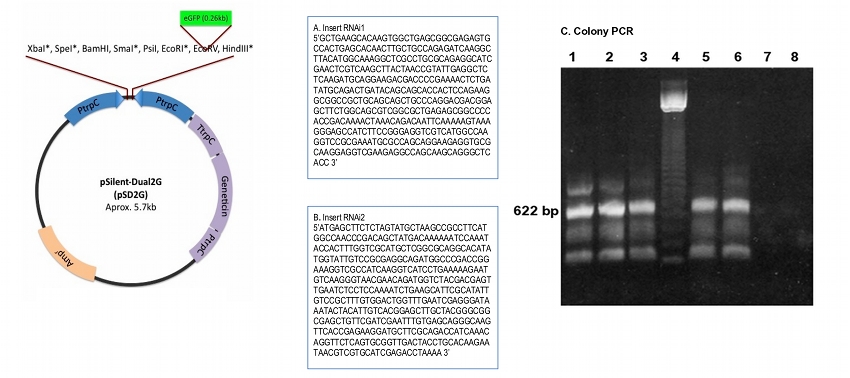

Supplement: Additional File 3 — pSD2G, sscmk1 inserts and colony PCR. This file shows pSD2G (pSD2G) from the Fungal Genetic Stock Center. It has a geneticin resistance cassette and two trpC promoters flanking the multiple cloning site (MCS). File 3A and 3B show the nucleotide sequences of the sscmk1 gene inserted into pSD2G: a 405 bp insert from the 3' region and a 432 bp insert from the 5' region of the gene. These inserts were amplified by PCR from cDNA containing the coding sequence of the sscmk1 gene, cloned in pCR®2.1-TOPO, excised by digestion with restriction enzymes and cloned in the MCS of pSD2G to produce pSD2G-RNAi1 and pSD2G-RNAi2, respectively. File 3C Shows the results of the colony PCR of various S. schenckii transformants. Cell suspensions of S. schenckii transformants were used as templates for PCR using the G418 (for)/G418 (rev) primer pair as described in Methods. Lane 4 shows the 123 bp DNA ladder. Lanes 1, 2, 3, 5 and 6 shows the bands obtained when the cells transformed with pSD2G-RNAi1 from colonies 14, 15, 18, 19 and 21 were used as template, respectively. In lanes 7 and 8, suspensions of non-transformed cells were used as templates for PCR. A band of the expected size, 622 bp, due to the presence of the geneticin resistance cassette was observed in transformed yeast cells. [file 1471-2180-11-162-S3.JPEG]
