# Supplementary material for: Calcium/calmodulin kinase1 and its relation to thermotolerance and HSP90 in Sporothrix schenckii: an RNAi and yeast two-hybrid study
Source: BMC Microbiol. 2011 Jul 11;11:162. doi: 10.1186/1471-2180-11-162 (PMC3146815; doi:10.1186/1471-2180-11-162)
Supplement: Additional File 4 — cDNA and derived amino acid sequence of the S. schenckii HSP90 homologue isolated using yeast two-hybrid assay. The cDNA and derived amino acid sequence of the SSHSP90 identified in the yeast two-hybrid assay as interacting with SSCMK1 is shown. Non-coding regions are given in lower case letters, coding regions and amino acids are given in upper case letters. The HATPase domain is shaded in yellow and the sequence isolated in the yeast two-hybrid assay is shaded in gray. Red letters mark the conserved MEEVD domain in the C terminal domain of HSP90, necessary for the interaction with tetratricopeptide repeat containing proteins. [file 1471-2180-11-162-S4.PDF]

```
   1 - aatttcattgtcagtctacgacactttccaccttttttccggacgacaaaacaaccagtaagcaccttagcatcttcttca - 81
  82 - agaaattatctgttctgccgtgaacagcatccgccaagATGTCTGGTGAAACTTTTGAGTTCCAGGCTGAGATCTCTCAGC - 162
   1 -                                      M  S  G  E  T  F  E  F  Q  A  E  I  S  Q  L  - 15
 163 - TCCTGTCTCTGATCATCAACACCGTCTACTCCAATAAGGAGATTTTCCTTCGGGAGCTTGTTTCCAATGCCTCTGATGCTC - 243
  16 -  L  S  L  I  I  N  T  V  Y  S  N  K  E  I  F  L  R  E  L  V  S  N  A  S  D  A  L  - 42
 244 - TGGACAAGATCCGGTACGAGTCATTGTCGGACCCCAGCAAGCTGGACTCTGGCAAAGACCTCCGCATCGACATCATCCCTG - 324
  43 -  D  K  I  R  Y  E  S  L  S  D  P  S  K  L  D  S  G  K  D  L  R  I  D  I  I  P  D  - 69
 325 - ACAAGGATGCCAAGACTCTTACGATCCGCGATACGGGTATCGGCATGACCAAGGCTGACCTCGTCAACAACCTCGGTACTA - 405
  70 -  K  D  A  K  T  L  T  I  R  D  T  G  I  G  M  T  K  A  D  L  V  N  N  L  G  T  I  - 96
 406 - TTGCTCGCTCGGGTACCAAGCAGTTCATGGAGGCCCTGACTGCCGGCGCCGATATCTCCATGATTGGCCAGTTCGGCGTCG - 486
  97 -  A  R  S  G  T  K  Q  F  M  E  A  L  T  A  G  A  D  I  S  M  I  G  Q  F  G  V  G  - 123
 487 - GTTTCTACTCGGCTTACCTGGTTGCCGACCAGGTTAAGGTCATCTCGAAGAACAACGACGACGAGCAGTACATCTGGGAGT - 567
 124 -  F  Y  S  A  Y  L  V  A  D  Q  V  K  V  I  S  K  N  N  D  D  E  Q  Y  I  W  E  S  - 150
 568 - CGAGTGCCGGCGGCACCTTCACCATCCTCCCGGACACGGAGGGTGAGCCCCTTGGCCGTGGTACGAAGATCATCCTGCACC - 648
 151 -  S  A  G  G  T  F  T  I  L  P  D  T  E  G  E  P  L  G  R  G  T  K  I  I  L  H  L  - 177
 649 - TAAAGGATGAGCAGATGGACTACCTTAACGAGAGCAAGGTTAAGGAGGTGATCAAGAAACATTCGGAGTTTATCTCCTATC - 729
 178 -  K  D  E  Q  M  D  Y  L  N  E  S  K  V  K  E  V  I  K  K  H  S  E  F  I  S  Y  P  - 204
 730 - CTATCTACCTCCACGTCAAGAAGGAGACTGAGAAGGAGGTTCCAGACGAGGATGCTGAGGAGGAAACTACTACTGAGGACT - 810
 205 -  I  Y  L  H  V  K  K  E  T  E  K  E  V  P  D  E  D  A  E  E  E  T  T  T  E  D  S  - 231
 811 - CCGACGATAAGAAGCCTAAGATTGAGGAGGTCAGCGACGATGAAGACGGCGAGGAGAAAGAGGACAAAAAGAAGAAGACAA - 891
 232 -  D  D  K  K  P  K  I  E  E  V  S  D  D  E  D  G  E  E  K  E  D  K  K  K  K  T  K  - 258
 892 - AGAAGGTGACGGAGACCACGATTGAAGAGGAGGAGCTGAACAAGCAGAAGCCCATCTGGACCCGCAACCCGCAGGACATAA - 972
 259 -  K  V  T  E  T  T  I  E  E  E  E  L  N  K  Q  K  P  I  W  T  R  N  P  Q  D  I  N  - 285
 973 - ACCAGGAGGAGTACGCTTCATTTTATAAGTCGCTCACAAACGACTGGGAGGACCACTTGGCCGTTAAGCACTTCTCGGTTG - 1053
 286 -  Q  E  E  Y  A  S  F  Y  K  S  L  T  N  D  W  E  D  H  L  A  V  K  H  F  S  V  E  - 312
1054 - AGGGTCAGCTCGAGTTCCGCGCTATCCTGTTTGTCCCGAAGCGCGCACCTTTCGATCTCTTTGAGACGAAGAAGACGAAGA - 1134
 313 -  G  Q  L  E  F  R  A  I  L  F  V  P  K  R  A  P  F  D  L  F  E  T  K  K  T  K  N  - 339
1135 - ACAACATCAAGCTCTACGTTCGCCGTGTCTTCATCACCGACGACGCTACTGATCTGGTTCCTGAGTGGCTAAGCTTTATCA - 1215
 340 -  N  I  K  L  Y  V  R  R  V  F  I  T  D  D  A  T  D  L  V  P  E  W  L  S  F  I  K  - 366
1216 - AGGGTGTCGTCGATTCTGAGGATCTTCCCCTGAACTTGTCTCGCGAGACACTGCAGCAGAACAAGATCATGAAGGTGATCA - 1296
 367 -  G  V  V  D  S  E  D  L  P  L  N  L  S  R  E  T  L  Q  Q  N  K  I  M  K  V  I  K  - 393
1297 - AGAAGAACATTGTGAAGAAGTCAATTGAGCTGTTCACTGAGATCTCCGAGGACAAGGAGCAATTCGACAAGTTCTACACGG - 1377
 394 -  K  N  I  V  K  K  S  I  E  L  F  T  E  I  S  E  D  K  E  Q  F  D  K  F  Y  T  A  - 420
1378 - CCTTCTCGAAGAACATCAAGCTGGGCATTCACGAGGACACCCAGAATCGCCCTGCGCTGGCCAAACTTCTGCGCTTCAATT - 1458
 421 -  F  S  K  N  I  K  L  G  I  H  E  D  T  Q  N  R  P  A  L  A  K  L  L  R  F  N  S  - 447
1459 - CAACCAAGTCGGGCGATGAGCAGACGTCGCTGGCCGACTATGTCACCCGGATGCCTGAGCACCAGAAGAACATGTACTACA - 1539
 448 -  T  K  S  G  D  E  Q  T  S  L  A  D  Y  V  T  R  M  P  E  H  Q  K  N  M  Y  Y  I  - 474
1540 - TCACTGGCGAGTCCATCAAGGCTGTGTCCCGTTCGCCTTTCCTTGACTCCCTAAAGGCCAAGGGCTTTGAGGTACTGTTCC - 1620
 475 -  T  G  E  S  I  K  A  V  S  R  S  P  F  L  D  S  L  K  A  K  G  F  E  V  L  F  L  - 501
1621 - TGGTCGACCCCATCGATGAATACGCCATGACACAGCTGAAGGAGTTTGAGGGCAAGAAGCTGGTTGACATCACGAAGGACT - 1701
 502 -  V  D  P  I  D  E  Y  A  M  T  Q  L  K  E  F  E  G  K  K  L  V  D  I  T  K  D  F  - 528
1702 - TCGAGCTTGAGGAAACCGACGAGGAGAAGAAGACCCGGGAGGCCGAGGAGAAGGAGTACGAGGGCGTCGCCAAGGCTCTGA - 1782
 529 -  E  L  E  E  T  D  E  E  K  K  T  R  E  A  E  E  K  E  Y  E  G  V  A  K  A  L  K  - 555
1783 - AGAATATCTTGGGCGACAAGGTCGAGAAGGTTGTTGTCTCCCACAAGCTGACGGGCTCGCCTTGCGCTATCCGTACCGGCC - 1863
 556 -  N  I  L  G  D  K  V  E  K  V  V  V  S  H  K  L  T  G  S  P  C  A  I  R  T  G  Q  - 582
1864 - AATTCGGCTGGTCTGCCAACATGGAGCGCATCATGAAGGCCCAGGCCCTGCGCGACACATCGATGTCGAGCTATATGTCGT - 1944
 583 -  F  G  W  S  A  N  M  E  R  I  M  K  A  Q  A  L  R  D  T  S  M  S  S  Y  M  S  S  - 609
1945 - CCAAGAAGACTTTTGAGATCTCTCCCCAGAGCCCGATCATCAAGGAACTCAAGAAGAAGGTTGAGGCCGACGGCGAGGACG - 2025
 610 -  K  K  T  F  E  I  S  P  Q  S  P  I  I  K  E  L  K  K  K  V  E  A  D  G  E  D  D  - 636
2026 - ACAAGACTGTCAAGTCCATCGTCCAGCTGCTGTTCGAGACATCTCTGCTGGTGTCTGGTTTCACCATCGACGAGCCTGCTA - 2106
 637 -  K  T  V  K  S  I  V  Q  L  L  F  E  T  S  L  L  V  S  G  F  T  I  D  E  P  A  S  - 663
2107 - GCTTTGCCGAGCGTATCCACAAGCTGGTCTCCCTCGGCCTGAACATTGATGAGGAGCCGGAGATCGACGACGCGGCTCCTA - 2187
 664 -  F  A  E  R  I  H  K  L  V  S  L  G  L  N  I  D  E  E  P  E  I  D  D  A  A  P  T  - 690
2188 - CTGAAACCCCGGCTGTTGCCGATGCTGGCGACAGCGCCATGGAGGAGGTTGACtaaaaccaccaaggcggaacgctccagc - 2268
 691 -  E  T  P  A  V  A  D  A  G  D  S  A  M  E  E  V  D  *                              - 707
2269 - tgacaaagagcgaggaggttgactaaaaccaccaaggctgttgcggtggatggataataaaatgaattggagttaggactg - 2349
2350 - gcctggctgtgttatttgcgttacatgatgcctcgtatttcgcgcgatttttttgatggatcaccccgacggcatgttttg - 2430
2431 - tcgacattctcacgatgttcagtgaacatgacttacgtggtatcattttacttgttgagtatgtagacctgcattaaggag - 2511
2512 - gtaattttccatggtgaaaaaaaaaaaaaaaaaaaaaaaaaa                                        - 2553
```
