# Supplementary material for: Calcium/calmodulin kinase1 and its relation to thermotolerance and HSP90 in Sporothrix schenckii: an RNAi and yeast two-hybrid study
Source: BMC Microbiol. 2011 Jul 11;11:162. doi: 10.1186/1471-2180-11-162 (PMC3146815; doi:10.1186/1471-2180-11-162)
Supplement: Additional File 5 — Amino acid sequence alignment of SSHSP90 to other fungal HSP90 homologues. The predicted amino acid sequence of S. schenckii SSHSP90 and HSP90 homologues from other fungi were aligned using M-Coffee. In the alignment, black shading with white letters indicates 100% identity, gray shading with white letters indicates 75-99% identity, gray shading with black letters indicates 50-74% identity. Important domains, the HATPase domain and theHSP 90 domain, are highlighted in blue and red boxes, respectively. The C terminal domain is indicated with a blue line. [file 1471-2180-11-162-S5.PDF]

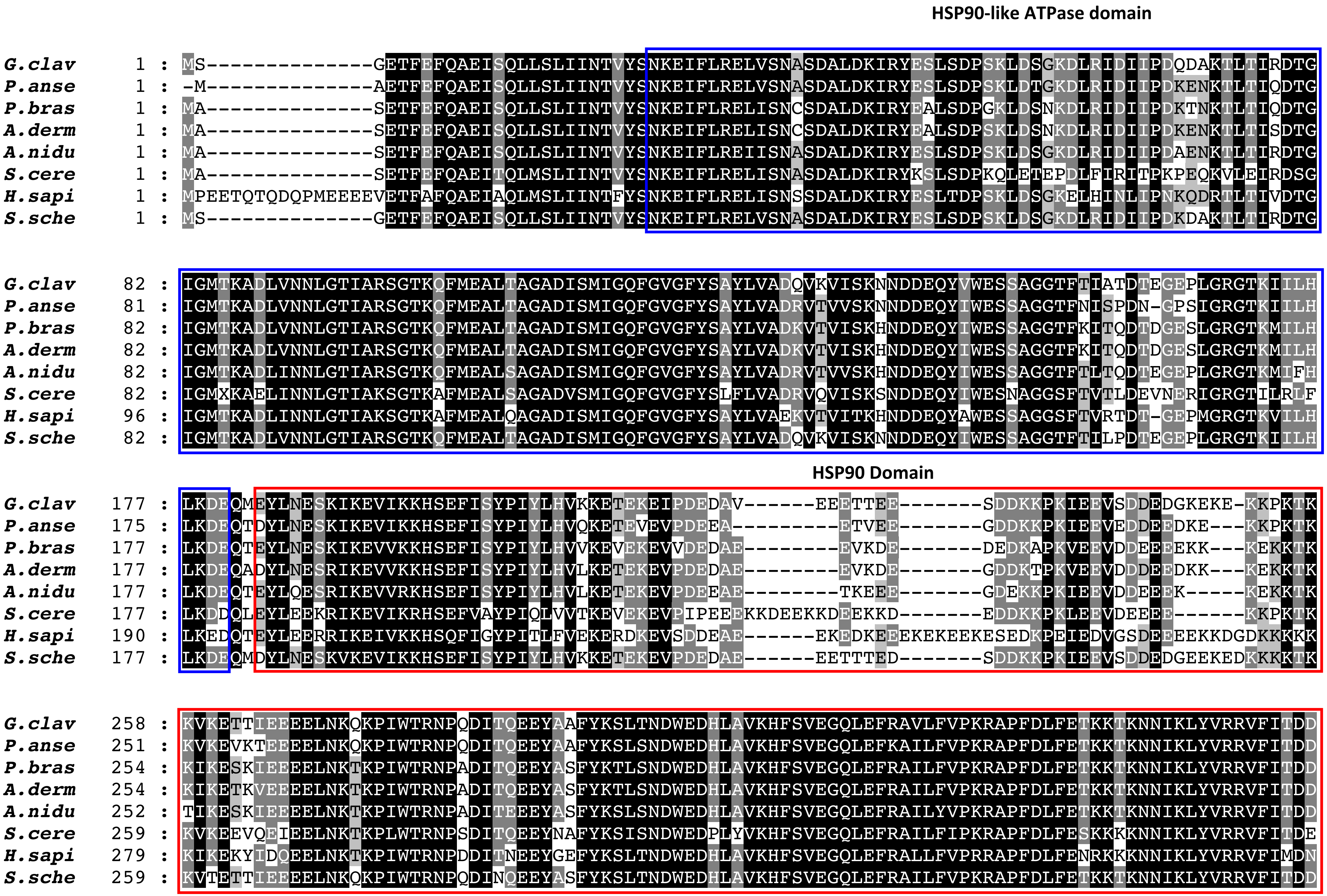
HSP90-like ATPase domain
G.clav 1 : MS---------------GETFEFQAEISQLLSLIINTVYSNKEIFLRELVSNASDALDKIRYESLSDPSKLDSGKDLRIDIIPDQDAKTLTIRDTG
P.anse 1 : -M---------------AETFEFQAEISQLLSLIINTVYSNKEIFLRELVSNASDALDKIRYESLSDPSKLDTGKDLRIDIIPDKENKTLTIQDTG
P.bras 1 : MA---------------SETFEFQAEISQLLSLIINTVYSNKEIFLRELISNCSDALDKIRYEALSDPGKLDSNKDLRIDIIPDKTNKTLTIQDTG
A.derm 1 : MA---------------SETFEFQAEISQLLSLIINTVYSNKEIFLRELISNCSDALDKIRYEALSDPSKLDSNKDLRIDIIPDKENKTLTISDTG
A.nidu 1 : MA---------------SETFEFQAEISQLLSLIINTVYSNKEIFLREIISNASDALDKIRYESLSDPSKLDSGKDLRIDIIPDAENKTLTIRDTG
S.cere 1 : MA---------------SETFEFQAEITQLMSLIINTVYSNKEIFLRELISNASDALDKIRYKSLSDPKQLETEPDLFIRITPKPEQKVLEIRDSG
H.sapi 1 : MPEETQTQDQPMEEEEVETFAFQAEIAQLMSLIINTFYSNKEIFLRELISNSSDALDKIRYESLTDPSKLDSGKELHINLIPNKQDRTLTIVDTG
S.sche 1 : MS---------------GETFEFQAEISQLLSLIINTVYSNKEIFLRELVSNASDALDKIRYESLSDPSKLDSGKDLRIDIIPDKDAKTLTIRDTG
G.clav 82 : IGMTKADLVNNLGTIARSGTKQFMEALTAGADISMIGQFGVGFYSAYLVADQVKVISKNNDDEQYVWESSAGGTFTIATDTEGEPLGRGTKIILH
P.anse 81 : IGMTKADLVNNLGTIARSGTKQFMEALTAGADISMIGQFGVGFYSAYLVADRVTVVSKNNDDEQYIWESSAGGTFNISPDN-GPSIGRGTKIILH
P.bras 82 : IGMTKADLVNNLGTIARSGTKQFMEALTAGADISMIGQFGVGFYSAYLVADKVTVISKHNDDEQYIWESSAGGTFKITQDTDGESLGRGTKMILH
A.derm 82 : IGMTKADLVNNLGTIARSGTKQFMEALTAGADISMIGQFGVGFYSAYLVADKVTVISKHNDDEQYIWESSAGGTFKITQDTDGESLGRGTKMILH
A.nidu 82 : IGMTKADLINNLGTIARSGTKQFMEALSAGADISMIGQFGVGFYSAYLVADRVTVVSKHNDDEQYIWESSAGGTFTLTQDTEGEPLGRGTKMIFH
S.cere 82 : IGMXKAELINNLGTIAKSGTKAFMEALSAGADVSMIGQFGVGFYSLFLVADRVQVISKSNDDEQYIWESNAGGSFTVTLDEVNERIGRGTILRLF
H.sapi 96 : IGMTKADLINNLGTIAKSGTKAFMEALQAGADISMIGQFGVGFYSAYLVAEKVTVITKHNDDEQYAWESSAGGSFTVRTDT-GEPMGRGTKVILH
S.sche 82 : IGMTKADLVNNLGTIARSGTKQFMEALTAGADISMIGQFGVGFYSAYLVADQVKVISKNNDDEQYIWESSAGGTFTILPDTEGEPLGRGTKIILH
HSP90 Domain
G.clav 177 : LKDEQMEYLNESKIKEVIKKHSEFISYPIYLHVKKETEKEIPDEDAV------EEETTEE-------SDDKKPKIEEVSDDEDGKEKE-KKPKTK
P.anse 175 : LKDEQTDYLNESKIKEVIKKHSEFISYPIYLHVQKETEVEVPDEEA---------ETVEE-------GDDKKPKIEEVEDDEEDKE---KKPKTK
P.bras 177 : LKDEQTEYLNESKIKEVVKKHSEFISYPIYLHVVKEVEKEVVDEDAE--------EVKDE-------DEDKAPKVEEVDDEEEEKK---KEKKTK
A.derm 177 : LKDEQADYLNESRIKEVVKKHSEFISYPIYLHVLKETEKEVPDEDAE--------EVKDE-------GDDKTPKVEEVDDDEEDKK---KEKKTK
A.nidu 177 : LKDEQTEYLQESRIKEVVRKHSEFISYPIYLHVLKETEKEVPDEEAE--------TKEEE-------GDEKKPKIEEVDDEEEK-----KEKKTK
S.cere 177 : LKDDQLEYLEEKRIKEVIKRHSEFVAYPIQLVVTKEVEKEVPIPEEEKKDEEKKDEEKKD-------EDDKKPKLEEVDEEEE------KKPKTK
H.sapi 190 : LKEDQTEYLEERRIKEIVKKHSQFIGYPITLFVEKERDKEVSDDEAE------EKEDKEEEKEKEEKESEDKPEIEDVGSDEEEEKKDGDKKKKKK
S.sche 177 : LKDEQMDYLNESKVKEVIKKHSEFISYPIYLHVKKETEKEVPDEDAE------EETTTED-------SDDKKPKIEEVSDDEDGEEKEDKKKKTK
G.clav 258 : KVKETTIEEEELNKQKPIWTRNPQDITQEEYAAFYKSLTNDWEDHLAVKHFSVEGQLEFRAVLFVPKRAPFDLFETKKTKNNIKLYVRRVFITDD
P.anse 251 : KVKEVKTEEEELNKQKPIWTRNPQDITQEEYAAFYKSLSNDWEDHLAVKHFSVEGQLEFKAILFVPKRAPFDLFETKKTKNNIKLYVRRVFITDD
P.bras 254 : KIKESKIEEEELNKTKPIWTRNPADITQEEYASFYKTLSNDWEDHLAVKHFSVEGQLEFRAILFVPKRAPFDLFETKKTKNNIKLYVRRVFITDD
A.derm 254 : KIKETKVEEEELNKTKPIWTRNPADITQEEYASFYKTLSNDWEDHLAVKHFSVEGQLEFRAILFVPKRAPFDLFETKKTKNNIKLYVRRVFITDD
A.nidu 252 : TIKESKIEEEELNKTKPIWTRNPADITEEEYASFYKSLSNDWEDHLAVKHFSVEGQLEFRAILYVPKRAPFDLFETKKTKNNIKLYVRRVFITDD
S.cere 259 : KVKEEVQEIEELNKTKPLWTRNPSDITQEEYNAFYKSISNDWEDPLYVKHFSVEGQLEFRAILFIPKRAPFDLFESKKKKNNIKLYVRRVFITDE
H.sapi 279 : KIKEKYIDQEELNKTKPIWTRNPDDITNEEYGEFYKSLTNDWEDHLAVKHFSVEGQLEFRALLFVPRRAPFDLFENRKKKNNIKLYVRRVFIMDN
S.sche 259 : KVTETTIEEEELNKQKPIWTRNPQDINQEEYASFYKSLTNDWEDHLAVKHFSVEGQLEFRAILFVPKRAPFDLFETKKTKNNIKLYVRRVFITDD

```
G.clav   353 : ATDLVPEWLSFVKGVVDSEDLPLNLSREILQQNKIMKVIKKNIVKKSIELFNEIAEDKEQFDKFYAAFSKNLKLGIHEDSQNRPALAKLLRFNST
P.anse   346 : ATDLIPEWLSFVKGVVDSEDLPLNLSRETLQQNKIMKVIRKNIVKKALELFTEIAEDKEQFDKFYTAFSKNIKLGIHEDSQNRNTLAKLLRFNST
P.bras   349 : ATDLIPEWLSFIKGVVDSEDLPLNLSRETLQQNKIMKVIKKNIVKKTLELFTEIAEDREQFDKFYSAFSKNIKLGIHEDAQNRPALAKLLRFNST
A.derm   349 : ATDLIPEWLSFIKGVVDSEDLPLNLSRETLQQNKIMKVIKKNIVKKTLELFNEIAEDREQFDKFYSAFSKNIKLGIHEDAQNRPALAKLLRFNST
A.nidu   347 : ATDLIPEWLGFVKGVVDSEDLPLNLSRETLQQNKIMKVIKKNIVKKTLELFNEIAEDREQFDKFYSAFSKNIKLGIHEDAQNRNTLAKLLRYQST
S.cere   354 : AEDLIPEWLSFVKGVVDSEDLPLNLSREMLQQNKIMKVIRKNIVKKLIEAFNEIAEDSEQFEKFYSAFSKNIKLGVHEDTQNRAALAKLLRYNST
H.sapi   374 : CEELIPEYLNFIRGVVDSEDLPLNISREMLQQSKILKVIRKNLVKKCLELFTELAEDKENYKKFYEQFSKNIKLGIHEDSQNRKKLSELLRYYTS
S.sche   354 : ATDLVPEWLSFIKGVVDSEDLPLNLSRETLQQNKIMKVIKKNIVKKSIELFTEISEDKEQFDKFYTAFSKNIKLGIHEDTQNRPALAKLLRFNST

G.clav   448 : KSGDELTSLSDYITRMPEHQKNIYYITGESIKAVTRSPFLDSLKEKGFEVLFLVDPIDEYAMTQLKEFESKKLVDITKD-FDLEETEEEKTSREA
P.anse   441 : KSGDEQTSLSDYVTRMPEHQKNMYYITGESIKAVSKSPFLDSLKEKGFEVLFLVDPIDEYAMTQLKEFEGKKLVDITKD-FELEETEEEKKQREA
P.bras   444 : KSGDETTSLADYVTRMQEHQKQMYYITGESLKAVQKSPFLDTLKEKNFEVLFLVDPIDEYAMTQLKEFDGKKLVDITKD-FELEETDEEKKTREA
A.derm   444 : KSGDETTSLADYVTRMQEHQKQMYYITGESLKAVQKSPFLDTLKEKNFEVLFLVDPIDEYAMTQLKEFDGKKLVDITKD-FELEETEEEKKAREA
A.nidu   442 : KSGDETTSLTDYVTRMKEHQKQIYYITGESIKAVAKSPFLDTLKQKDFEVLFLVDPIDEYAFTQLKEFDGKKLVDITKD-FELEETDEEKAEREK
S.cere   449 : KSVDELTSLTDYVTRMPEHQKNIYYITGESLKAVEKSPFLDALKAKNFEVLFLTDPIDEYAFTQLKEFEGKTLVDITKD-FELEETDEEKAEREK
H.sapi   469 : ASGDEMVSLKDYCTRMKENQKHIYYITGETKDQVANSAFVERLRKHGLEVIYMIEPIDEYCVQQLKEFEGKTLVSVTKEGLELPEDEEEKKKQEE
S.sche   449 : KSGDEQTSLADYVTRMPEHQKNMYYITGESIKAVSRSPFLDSLKAKGFEVLFLVDPIDEYAMTQLKEFEGKKLVDITKD-FELEETDEEKKTREA

G.clav   542 : EEKEYEGLAKALKNILGDKVEKVVVSHKLVGAPCAIRTGQFGWSANMERIMKAQALRDTSMSSYMSSKKTFEISPKNSIIKELKKKVETDGEDDK
P.anse   535 : EEKEYDGLAKALKNVLGDKVEKVVVSHKLVGAPCAIRTGQFGWSANMERIMKAQALRDTSMSSYMSSKKTFEISPKSPIIKELKQKVEADGENDK
P.bras   538 : EEKEFEGLAKALKNVLGDKVEKVVVSHKLIGSPCAIRTGQFGWSANMERIMKAQALRDTSMSSYMSSKKTFEISPRSPIIKELKKKVEADGENDR
A.derm   538 : EEKEYEGLAKSLKNVLGDKVEKVVVSHKLIGSPCAIRTGQFGWSANMERIMKAQALRDTSMSSYMSSKKTFEISPKSPIIQELKKKVEADGENDR
A.nidu   536 : EEKEFENLAKSLKNILGDKVEKVVVSHKLIGSPCAIRTGQFGWSANMERIMKAQALRDTSMSSYMSSKKTFEISPKSPIIKELKKKVEADGESDR
S.cere   543 : EIKEYEPLTKALKEILGDQVEKVVVSYXLLDAPAAIRTGQFGWSANMERIMKAQALRDSSMSSYMSSKKTFEISPKSPIIKELKKRVDEGGAQDK
H.sapi   564 : KKTKFENLCKIMKDILEKKVEKVVVSNRLVTSPCCIVTSTYGWTANMERIMKAQALRDNSTMGYMAAKKHLEINPDHSIIETLRQKAEAD-KNDK
S.sche   543 : EEKEYEGVAKALKNILGDKVEKVVVSHKLTGSPCAIRTGQFGWSANMERIMKAQALRDTSMSSYMSSKKTFEISPQSPIIKELKKKVEADGEDDK

G.clav   637 : TVKSIVQLLFETSLLVSGFTIDEPASFSERIHKLVSLGLNIDEEPET--E---AAPTDAGASAAET--GD-SAMEEVD
P.anse   630 : TVKSIVQLLFETSLLVSGFTIEEPAGFAERIHKLVALGLNLDEEPEAAAD---APAADAGVAAAET--SD-NAMEEVD
P.bras   633 : TVKSITQLLFETSLLVSGFTIEEPAGFAERIHKLVSLGLNVDEESEKE-GGDSTDKEAAPAAAELA--GE-SAMEEVD
A.derm   633 : TVKSITQLLFETSLLVSGFTIEEPAGFAERIHKLVSLGLNIDEEAETT---EEKETEEAAAPAEVA--GE-SAMEEVD
A.nidu   631 : TVKSITQLLYETSLLVSGFTIEEPASFAERIHKLVSLGLNIDEEAEA-----EPASTEEAPAAATT--GE-SAMEEVD
S.cere   638 : TVKDLTKLLYETALLTSGFSLDEPTSFASRINRLISLGLNIDEDEETE-T--APEASTAAPVEEVP--AD-TEMEEVD
H.sapi   658 : SVKDLVILLYETALLSSGFSLEDPQTHANRIYRMIKLGLGIDEDDPTA--D-DTSAAVTEEMPPLEGDDDTSRMEEVD
S.sche   638 : TVKSIVQLLFETSLLVSGFTIDEPASFAERIHKLVSLGLNIDEEPEI--D---DAAPTETPAVADA--GD-SAMEEVD
```
